# Transferrin-Conjugated PLGA Nanoparticles for Co-Delivery of Temozolomide and Bortezomib to Glioblastoma Cells

**DOI:** 10.1021/acsanm.3c02122

**Published:** 2023-08-03

**Authors:** Maria João Ramalho, Inês David Torres, Joana Angélica Loureiro, Jorge Lima, Maria Carmo Pereira

**Affiliations:** † LEPABE—Laboratory for Process Engineering, Environment, Biotechnology and Energy, Faculty of Engineering, University of Porto, Rua Dr. Roberto Frias, 4200-465 Porto, Portugal; ‡ ALiCE—Associate Laboratory in Chemical Engineering, Faculty of Engineering, University of Porto, Rua Dr. Roberto Frias, 4200-465 Porto, Portugal; § i3S—Instituto de Investigação e Inovação em Saúde, Universidade do Porto, R. Alfredo Allen, 4200-10 135 Porto, Portugal; ∥ Ipatimup—Instituto de Patologia e Imunologia Molecular da Universidade do Porto, Rua Júlio Amaral de Carvalho 45, 4200-135, Porto, Portugal; ⊥ Faculty of Medicine of Porto University, Alameda Prof. Hernâni Monteiro, 4200-319, Porto, Portugal

**Keywords:** high-grade glioma, brain tumor, MGMT protein, drug resistance mechanisms, alkylating agents, poly(lactic-*co*-glycolic acid), brain delivery, transferrin receptor

## Abstract

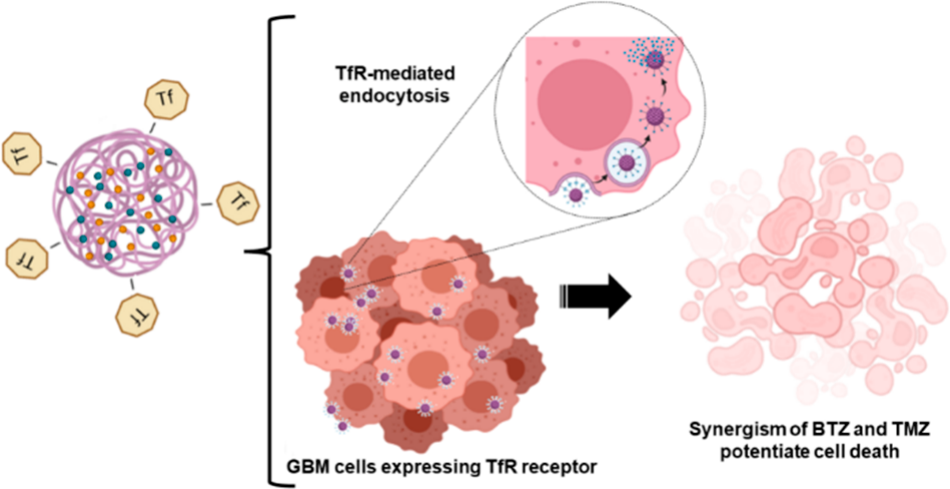

Glioblastoma (GBM) represents almost half of primary brain tumors, and its standard treatment with the alkylating agent temozolomide (TMZ) is not curative. Treatment failure is partially related to intrinsic resistance mechanisms mediated by the O6-methylguanine-DNA methyltransferase (MGMT) protein, frequently overexpressed in GBM patients. Clinical trials have shown that the anticancer agent bortezomib (BTZ) can increase TMZ’s therapeutic efficacy in GBM patients by downregulating MGMT expression. However, the clinical application of this therapeutic strategy has been stalled due to the high toxicity of the combined therapy. The co-delivery of TMZ and BTZ through nanoparticles (NPs) of poly(lactic-*co*-glycolic acid) (PLGA) is proposed in this work, aiming to explore their synergistic effect while decreasing the drug’s toxicity. The developed NPs were optimized by central composite design (CCD), then further conjugated with transferrin (Tf) to enhance their GBM targeting ability by targeting the blood–brain barrier (BBB) and the cancer cells. The obtained NPs exhibited suitable GBM cell delivery features (sizes lower than 200 nm, low polydispersity, and negative surface charge) and a controlled and sustained release for 20 days. The uptake and antiproliferative effect of the developed NPs were evaluated in *in vitro* human GBM models. The obtained results disclosed that the NPs are rapidly taken up by the GBM cells, promoting synergistic drug effects in inhibiting tumor cell survival and proliferation. This cytotoxicity was associated with significant cellular morphological changes. Additionally, the biocompatibility of unloaded NPs was evaluated in healthy brain cells, demonstrating the safety of the nanocarrier. These findings prove that co-delivery of BTZ and TMZ in Tf-conjugated PLGA NPs is a promising approach to treat GBM, overcoming the limitations of current therapeutic strategies, such as drug resistance and increased side effects.

## 1 Introduction

Glioblastoma (GBM) is the most frequent and invasive form of brain tumors. These tumors have a poor response to the currently available therapies, being associated with the rapid evolution and fatal prognosis with a median survival of 16 months.^1^ GBM standard of care is the Stupp protocol and includes neurosurgery for tumor resection followed by radiotherapy cycles combined with chemotherapy with temozolomide (TMZ).^2^ TMZ is an alkylating agent that damages DNA, inhibiting cell replication and consequently leading to cell death. However, chemotherapy with TMZ is associated with frequent side effects due to its high toxicity. Also, the active metabolite of TMZ possesses low permeability through biological membranes, such as the membrane of tumor cells and the blood–brain barrier (BBB), which decreases its bioavailability and accumulation in the target tumor tissue.^3^

Additionally, the success of chemotherapy with TMZ or other alkylating agents is limited by resistance mechanisms, such as those mediated by the O6-methylguanine-DNA methyltransferase (MGMT) protein. This enzyme confers resistance to therapy by repairing the TMZ-induced DNA damage, and it is reported to be overexpressed in 40–60% of GBM patients.^4^ Thus, MGMT has attracted interest as a therapeutic target in combination with TMZ.

In recent years different strategies have been explored to circumvent the MGMT resistance, such as the modulation of MGMT expression and/or transcription or directly inactivating the MGMT protein.^5^ Different drugs with potential MGMT inhibitory effects have been studied and aligned with the recent investment in drug repurposing strategies for GBM, aiming to identify new uses for already approved drugs.^6^

One such drug is bortezomib (BTZ), an FDA-approved drug for multiple myeloma treatment^7^ that can downregulate MGMT protein expression, leading to increased efficacy of TMZ in GBM patients.^8,9^ BTZ blocks the activity of κ-light-chain-enhancer of activated B cells factor (NF-κB), downregulating the MGMT gene expression and enhancing the sensitivity of GBM to TMZ.^10^

However, studies have reported that the systemic coadministration of MGMT inhibitors with TMZ or other alkylating agents is associated with toxicity to the healthy tissues, particularly inducing hematopoietic toxicity and myelosuppression, due to the deficient MGMT expression in hematopoietic stem cells.^11,12^ Thus, BTZ accumulation in healthy organs should be avoided to prevent an exacerbated toxicity of TMZ. Additionally, the independent systemic administration of two drugs with different pharmacokinetics and biodistribution might lead to an ineffective therapeutic outcome due to variations in their molar ratio over time.^13^ Indeed, pharmacokinetic parameters of TMZ and BTZ are significantly different, such as peak concentration (for TMZ *C*_max_ = 7000 ng/mL; for BTZ *C*_max_ = 223 ng/mL), time to *C*_max_ (for TMZ *t*_max_ ≈ 1 h; for BTZ *t*_max_ ≈ 0.3 h), and clearance rates (5.5 and 7.79 (L/h)/m^2^ for TMZ and BTZ, respectively), as reported by the European Medicines Agency (EMA).

Polymeric nanoparticles for drug delivery have been widely explored to overcome the typical limitations of drugs’ systemic administration, as these can decrease systemic toxicity while increasing bioavailability in the target tissues.^14−16^ Thus, in this work, we tested the coencapsulation of TMZ and BTZ in biodegradable, biocompatible, and FDA-approved poly(lactic-*co*-glycolic acid) (PLGA) nanoparticles (NPs). Recently, our group evaluated the safety of PLGA NPs for long-term *in vivo* administration.^17^ A repeated-dose toxicity study was conducted with C57BL/6 mice, and the animals were administered bare PLGA NPs for 56 days (in a total of 24 injections per animal, at 100 mg/kg). All treated animals revealed no clinical signs of toxicity, demonstrating that these NPs are safe for repeated-dose exposure and can be used for BTZ and TMZ co-delivery for GBM therapy. Although several nanocarriers have been developed for TMZ delivery in recent years,^18^ this is the first time that the coencapsulation of TMZ and BTZ has been reported.

The production of the TMZ+BTZ PLGA NPs was optimized by experimental design, and to further increase the brain targeting ability of the proposed NPs, these were conjugated with transferrin (Tf) since the Tf receptor (TfR) is overexpressed in the blood–brain barrier (BBB) and GBM cells.^19,20^ After physicochemical characterization, the therapeutic effect of the developed NPs was evaluated by using human GBM cells with different MGMT expressions. The biocompatibility of the materials was also confirmed in healthy brain cells (immortalized human astrocytes).

## 2 Materials and Methods

### 2.1 Chemicals

Polyvinyl alcohol 4-88 (Mowiol 4-88, MW 31 000) (PVA), dichloromethane (MW 84.93; purity = 99%) (DCM), ethyl acetate (99.6%, MW 88.11), dimethyl sulfoxide (DMSO) (≥99.9%, MW 78.13), acetic acid (≥98%, MW 60.05), PLGA (Resomer RG 503 H, 50:50; MW 24 000–38 000), holo-transferrin human (MW 80.00, purity ≥98%), *N*-(3-dimethylaminopropyl)-*N*′-ethyl carbodiimide hydrochloride (EDC) (MW 191.70), phosphate-buffered saline (PBS), Pierce BCA protein assay kit, sulforhodamine B (SRB) (MW 580.66), trichloroacetic acid (TCA) (99%, MW 163.38), and tris(hydroxymethyl)aminomethane (≥99.8%, MW 141.14) were purchased from Sigma-Aldrich. TMZ (MW 194.15, purity ≥99%) and BTZ (MW 384.24, purity >99%) were acquired from Selleck Chemicals (Munich, Germany). Trypan blue (≥70%, MW 960.80) was obtained from Biochem Chemopharma (Cosne-Cours-sur-Loire, France). Trypsin (Gibco TrypLE) and fetal bovine serum (Gibco FBS) were purchased from Fisher Scientific (Hampton, NH, USA). High-glucose Dulbecco’s modified Eagle medium (DMEM) was obtained from Capricorn Scientific (Ebsdorfergrund, Germany). Penicillin–streptomycin and fungizone were acquired from Biowest LCC (Riverside, MO, USA).

### 2.2 Cells

The U251 and T98G human GBM cells were used in this work due to their different MGMT expression. U251 cells were selected due to their high sensitivity to TMZ’s effect and low MGMT expression,^21^ and T98G was chosen due to being TMZ-resistant due to high MGMT endogenous levels.^21^ An immortalized human astrocyte cell line (NHA) was used as a control due to their low expression of TfR when compared with GBM cells.^22^ The cell culture was maintained in DMEM medium containing 10% FBS, 1% fungizone, and 1% penicillin–streptomycin. Cells were trypsinized and passaged at a confluence of approximately 80%. During all of the experimental work, the cells were kept in a humidified 5%CO_2_ incubator at 37 °C.

### 2.3 Synthesis of PLGA NPS Containing BTZ+TMZ

**Figure 1:**
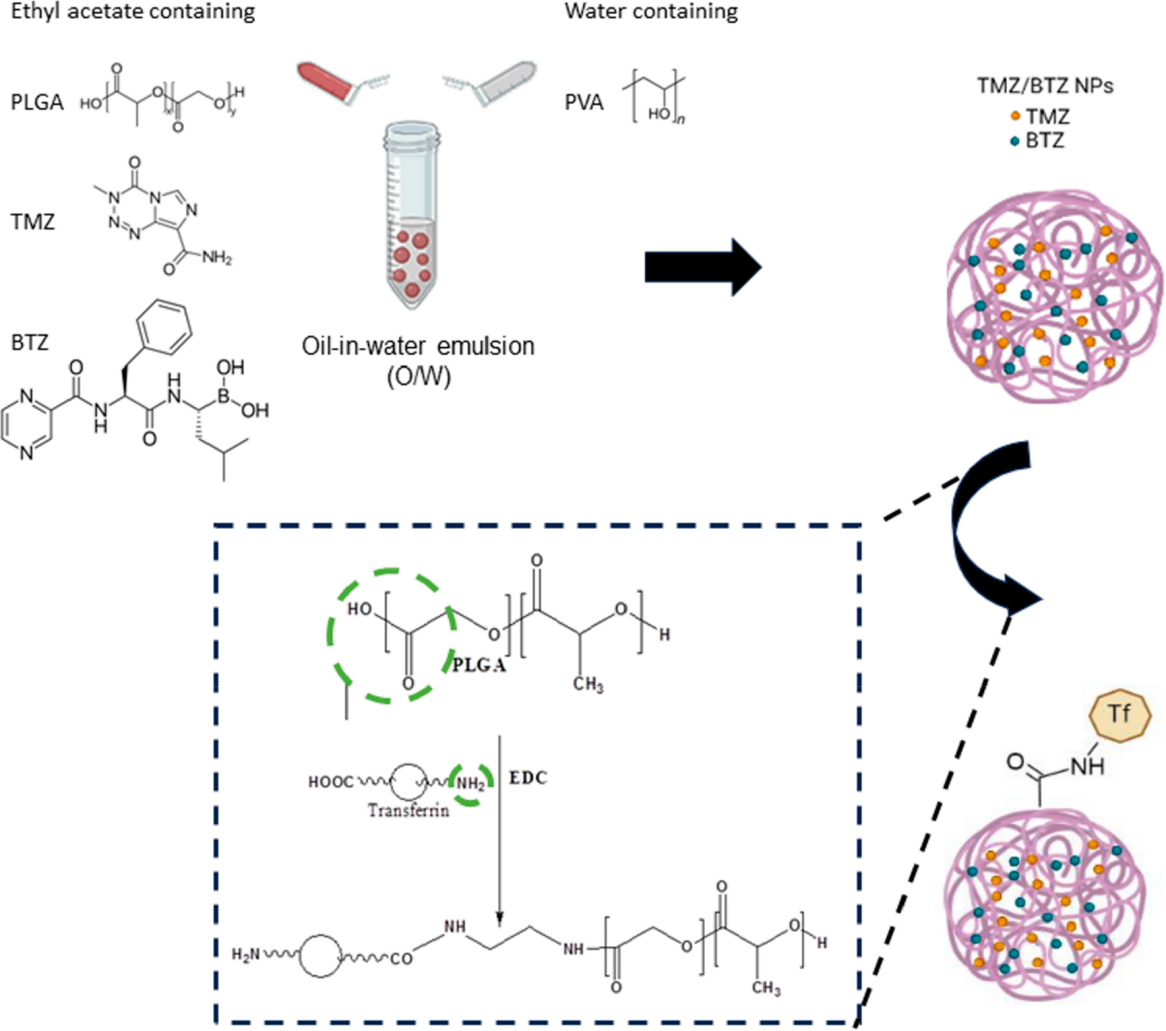
Synthesis of PLGA NPs containing TMZ and BTZ and their conjugation with Tf by the EDC click reaction.

The single emulsion-solvent evaporation method was applied to prepare PLGA NPs containing BTZ and TMZ (Figure 1). First, 0.5 mg of TMZ, 0.5 mg of BTZ, and PLGA were dissolved in ethyl acetate. Then, 2.0 mL of a PVA aqueous solution was added dropwise to the previously prepared organic solution The sample was emulsified by agitation (vortex, Genius 3, ikavortex, Germany) and then sonicated (UP400S ultrasonic processor, Hielscher, Berlin, Germany). Sonication cycles of 10 s on/off each at 40% amplification and an ultrasonic frequency of 24 kHz were applied. The number of cycles was modified. The preparation protocol was optimized by implementing an experimental design. After model validation, it was determined that optimal experimental parameters for NPs production were 19 mg of PLGA, 1.26% (w/v) of PVA, 4 sonication cycles, and a 0.667 O/W ratio. For detailed information, see the Supporting Information.

The samples were agitated at 800 rpm (Colosquid, ika, magnetic stirrer) to promote solvent evaporation. To recover the NPs, a centrifugation steps sequence was performed with increasing speed (from 5000 to 14 500 rpm) and duration (from 2 to 15 min). The NPs were resuspended in 1.0 mL of ultrapure water for subsequent analysis, and the supernatant was saved for nonencapsulated drug quantification. For cell experiments, NPs were produced under sterile conditions.

### 2.4 Encapsulation Efficiency and Loading Capacity

The NPs’ loading capacity (LC) and encapsulation efficiency (EE) were obtained indirectly by quantifying the unloaded drug in the supernatant.

Free TMZ was quantified by fluorescence at excitation and emission wavelengths of 420 and 540 nm, respectively (Synergy 2 microplate reader, BioTek, UK). UV–vis absorbance measurements quantified free BTZ at λ_max_ = 269 nm (Synergy 2 microplate reader, BioTek, U.K.). The obtained absorbance and fluorescence values were correlated to the BTZ and TMZ calibration curves in PVA, respectively.

The EE values were expressed as the percentage of the entrapped drug to the total used drug. The LC of the NPs was given as the percentage of the entrapped drugs to the PLGA weight.

### 2.5 Functionalization of NPS

The TMZ+BTZ-loaded PLGA NPs were further conjugated with transferrin (Tf) by a carbodiimide coupling reaction. In this reaction, the polymer carboxylic groups are first activated by EDC to bind to the primary amines of Tf, leading to the formation of an amide bond in a reaction known as a chemical click reaction.^23^

An EDC aqueous solution was added to the NPs’ suspension at a 20× molar excess, and the sample was continuously agitated at room temperature for 30 min. Then, an aqueous solution of Tf was added to the NPs (2× molar excess). The final goal was to obtain around 100 molecules of Tf per NP. The prepared solution was continuously agitated for 1 h at room temperature. After 1 h, the Tf-conjugated NPs were collected by centrifugation and resuspended in ultrapure water. The supernatant containing the unbounded Tf was saved to determine the conjugation efficiency (CE) using the bicinchoninic acid (BCA) protein assay.^24^ Briefly, the supernatant was incubated with the BCA kit reagent at a ratio of 1:20 (v/v) for 30 min at 37 °C, and UV–vis absorbance at λ_max_ = 562 nm was measured (Synergy 2 microplate reader, BioTek, U.K.) and correlated to a Tf calibration curve.

The presence of Tf on the PLGA NPs surface was further investigated by using attenuated total reflectance (ATR) Fourier transform infrared (FTIR) spectroscopy. All samples in aqueous suspension were dried, and spectra were recorded in absorbance mode in the 4000–350 cm^–1^ spectral range using a Bruker spectrometer Alpha (Bruker Optics Inc., Billerica, MA, USA) equipped with a platinum diamond crystal and a DTGS detector at a resolution of 4 cm^–1^.

### 2.6 NPS Physicochemical Characterization

Dynamic light scattering (DLS) was used to determine the average diameter, size distribution (PDI), and ζ potential of the prepared NPs by using a ZetaSizer Nano ZS (Malvern Instruments, U.K.). Size and PDI analyses were performed in a standard cuvette (Sarstedt) made of polystyrene, applying the dielectric constant of water as a dispersant.^25^ The sample was diluted (1:10) in ultrapure water to a final NPs concentration of 1 mg/mL. The Smoluchowski model using a DTS1070 cell was employed to determine the ζ potential values. DLS measurements were also conducted for 10 weeks to assess variations of the size, PDI, and ζ potential in storage conditions (aqueous suspension, pH 7.0; 4 °C) of the nonconjugated and Tf-conjugated TMZ+BTZ loaded PLGA NPs.

Transmission electron microscopy (TEM) was used for the morphological characterization of the developed NPs. Briefly, the NPs were soaked on copper grids (Formvar/carbon-400 mesh copper, Agar Scientific, U.K.) for negative staining with 2% (v/v) uranyl acetate in water and air-dried before visualization (Jeol JEM 1400 electron microscope, accelerating voltage of 80 kV, Japan).^26^

### 2.7 *In Vitro* Release of BTZ and TMZ

*In vitro* studies to evaluate the release of BTZ and TMZ from the nonmodified and Tf-modified BTZ+TMZ coloaded NPs were performed in two simulated physiological conditions over 20 days. Two different pH values were used to simulate healthy brain tissue/blood circulation and the GBM tumor microenvironment (6.4 for tumor tissue and 7.4 for blood/brain tissue).

Briefly, the NPs were resuspended in a total volume of 6 mL of PBS (0.01 M, NaCl 0.138 M; KCl 0.0027 M) at 37 °C and divided into 12 aliquots (0.5 mL each). 0.1 M HCl was used to adjust the PBS pH. The samples were kept at continuous gentle agitation (100 rpm) to simulate the *in vivo* movement of body fluids. At each time point, the NPs’ aliquot samples were centrifuged to separate the NPs from the supernatant containing the released drugs. The released drugs were quantified by fluorescence intensity measurements for TMZ and UV–vis absorbance for BTZ (Synergy 2 microplate reader, BioTek, U.K.) and correlated to control samples for each drug. Then TMZ and BTZ release curves were plotted as the percentage of compounds released as a function of time.

DLS measurements were also conducted for the duration of the experiment (20 days) to assess variations of the size, PDI, and ζ potential in both the release buffers (aqueous suspension, pH 7.0; 4 °C) of the nonconjugated and Tf-conjugated TMZ+BTZ loaded PLGA NPs.

#### 2.7.1 *In Vitro* Conformational Studies of TF

To evaluate if Tf suffers conformational changes under acidic conditions encountered in the release experiments, circular dichroism (CD) and ATR-FTIR techniques were employed. Experiments were performed at different pHs (7.4, 6.4, and 3.8). pH 3.8 corresponds to the pH measured at the end of the release experiments due to the accumulation of NPs’ acidic degradation products. Near UV-CD experiments were performed to evaluate the tertiary structure of Tf. Spectra were recorded on a JASCO J-815 spectropolarimeter (JASCO Corporation, Tokyo, Japan). Briefly, 120 μL of samples was placed on a 10 mm light path length quartz cell. Spectra were recorded at 20 °C between 250 and 320 nm with a bandwidth of 1 nm, response of 2 s, and scanning rate of 50 nm/min. Each resulting spectrum corresponds to an average of 16 scans. Spectra of the background were collected and subtracted from the corresponding protein spectrum. The results were converted to molar ellipticity, [θ], by normalizing the ellipticity to path length (*l*) and molar concentration (*c*) of each sample, using the following equation:
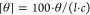
1

The secondary structure of Tf was further evaluated by ATR-FTIR. All samples in aqueous suspension were dried, and spectra were recorded in absorbance mode in the 4000–350 cm^–1^ spectral range (Bruker spectrometer Alpha, Bruker Optics Inc., Billerica, MA, USA). Data were treated using Origin 2021 software (OriginLab Corp., MA, USA). Fourier self-deconvolution algorithm with a smoothing factor of 0.4 was applied, followed by a baseline correction. Spectra deconvolution was performed using the Gaussian–Lorentzian function.

### 2.8 Cell Experiments

#### 2.8.1 Cell Uptake Assay

##### 2.8.1.1 Fluorescence Quantification Studies

Fluorescence measurements were used to quantify the cell uptake of Tf-conjugated and nonconjugated NPs. For that purpose, the NPs were labeled with coumarin-6 (C6) by loading C6 using the single-emulsion evaporation method.^27^ Nonloaded C6 was separated from the C6-loaded NPs by centrifugation. For the experiments, 8000 U251, T98G, or NHA cells were seeded in 96-well plates and left to adhere for 24 h. Then, Tf-conjugated and nonconjugated C6-PLGA NPs diluted in DMEM at a final PLGA concentration of 20 μM (100 μL) were added to the cells. Two-period treatments were tested to assess if the NPs internalization was a time-related process: 30 min and 120 min. At the end of the experiment, the cells were washed with PBS to remove the NPs that were not taken up by the cells. The cells were disrupted with 0.1% Triton X-100 in 0.1 M NaOH to allow for fluorescence quantification. The fluorescence of C6-NPs was determined at 430/485 nm excitation/emission wavelengths (microplate reader, BioTek Synergy 2, BioTek, U.K.).

##### 2.8.1.2 Competitive Binding to the TFR

A competitive receptor-blocking experiment was performed to further evaluate the TfR’s function in the NPs’ uptake mechanism. An excess of Tf was used to block the TfR before treatment with 100 μL of 20 μM C6-loaded PLGA NPs. Briefly, 8000 cells of U251, T98G, and NHA lines were seeded in 96-well plates. After 24 h for cell adhesion, the cells were incubated for 1 h with Tf solution in DMEM at concentrations between 1 and 6 mg.mL^–1^ (100 μL). After removing the unbound Tf, Tf-conjugated and nonconjugated C6-PLGA NPs diluted in DMEM (20 μM, (100 μL) were added to the cells. After 2 h of incubation, the cells were prepared for fluorescence measurements, as mentioned in the prior section.

#### 2.8.2 Cell Viability

##### 2.8.2.1 SRB Method for Combined Therapy

For the cytotoxicity evaluation, 1000 cells/well of U251 or T98G lines were seeded in 96-well plates and left to adhere for 24 h. After, the cells were treated for 72 h with 100 μL of free TMZ (10 nM to 2.0 × 10^6^ nM), free BTZ (1.0 × 10^–4^ to 2.0 × 10^3^ nM), or free TMZ+BTZ. A constant TMZ:BTZ ratio (1:0.8) was used for the combined therapy, with concentrations ranging between 1.0 × 10^–5^ and 5.0 × 10^2^ for TMZ, 8.0 × 10^–6^ and 4.0 × 10^2^ nM for BTZ. The studied TMZ:BTZ ratio was chosen based on both drugs’ EE (%) in the developed NPs. Untreated cells were used as the negative control. After treatment, the cells were fixed with trichloroacetic acid (TCA) 10% (w/v) at 4 °C for 1 h, then washed with ultrapure water and dried at room temperature. Then, cells were stained with SRB for 20 min. The samples were washed twice with 1% (v/v) acetic acid and air-dried to remove the unbound dye. The protein-bound dye was solubilized by a Tris buffer solution. The cell protein quantification was measured by UV–vis absorbance (BioTek Synergy 2 microplate reader, BioTek, U.K.) at 560 nm. Cell survival inhibition as a function of drug concentration was then plotted using GraphPad 9.3.1 software (GraphPad Software Inc., USA).

##### 2.8.2.2 Combination Index Determination

To evaluate whether the cytotoxic effect of the combined therapy of BTZ and TMZ in GBM cells (U251 and T98G cells) was synergistic or not, the combination index (CI) was calculated using the Chou–Talalay method.^28^ Then, the CompuSyn 1.0 software (The ComboSyn, Inc., USA) was used to determine the CI value by applying the following equation:^29^
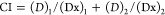
2where (Dx)_1_ and (Dx)_2_ represent the individual doses of TMZ and BTZ, respectively, that in combination give the same response as TMZ alone (*D*)_1_ and BTZ alone (*D*)_2_. CI values below 1 reveal synergism, values equal to 1 reveal additive effect, and values above 1 show antagonism.

##### 2.8.2.3 Antitumor Activity Assessment of Drug Loaded NPS

The SRB colorimetric assay was also employed to evaluate the antiproliferative effect of the combination of TMZ+BTZ loaded in Tf-conjugated and nonconjugated NPs in U251 and T98G cells. 1000 cells/well were seeded in 96-well plates and left to adhere for 24 h. Then, Tf-conjugated and nonconjugated TMZ+BTZ NPs were diluted in DMEM, and 100 μL of NPs suspensions were added to the cells at TMZ concentrations between 1.0 × 10^–5^ and 5.0 × 10^2^ nM, and BTZ final concentrations between 8.0 × 10^–6^ and 4.0 × 10^2^ nM (TMZ:BTZ ratio 1:0.8). Untreated cells were used as the negative control. After 72 h of treatment, an SRB assay was employed to evaluate cell viability, as described above. Cell survival inhibition as a function of drug concentration was then plotted using GraphPad 9.3.1 software (GraphPad Software Inc., USA). The IC_50_ values (the concentration inhibiting the cell survival by 50%) were obtained from the nonlinear regression of the dose–response curves.

#### 2.8.3 Biocompatibility Evaluation of NPS

The biocompatibility of the unloaded Tf-conjugated and nonconjugated NPs was evaluated in both U251 and T98G GBM cells and a human immortalized astrocyte cell line NHA as control. Briefly, 1000 cells/well were seeded in 96-well plates and left to adhere for 24 h. 100 μL of Tf-conjugated and nonconjugated NPs bare PLGA NPs were added to the cells in two final PLGA concentrations (5 μM and 5 mM). After 72 h, cell viability was assessed by the SBR assay as described above. Nontreated cells were also included as the negative control.

#### 2.8.4 Morphological Analysis

The cells were fixed with 10% TCA after the treatments mentioned above for the morphological analysis. The cells were then visualized and photographed with an Eclipse Ti–S inverted fluorescence microscope (Nikon, Carnaxide, Portugal). All photos were taken at 100× magnification.

### 2.9 Statistical Analysis

All the results are presented as the mean ± standard deviation (SD) for three independent experiments. The Student *t* test was applied for the statistical analysis, with a 95% confidence interval, *p* < 0.05 being considered significant.

## 3 Results and Discussion

### 3.1 Physicochemical Properties of TF-Conjugated BTZ+TMZ NPS

Optimized BTZ+TMZ NPs were produced after the validation of the experimental design (for more information, see the Supporting Information). The optimized TMZ+BTZ loaded PLGA NPs were then further conjugated with human holo-Tf for an active targeting delivery approach by a carbodiimide-coupling reaction. Tf was chosen as the targeting ligand since the TfR expression on GBM cells is about 100-fold higher than on healthy cells.^30^ The use of Tf molecules over antibodies is advantageous due to being nonimmunogenic and easily obtained from human sources at a relatively low cost.^31^ In this work, holo-Tf was selected due to having a superior affinity for the TfR than apo-Tf.^32^

**Figure 2:**
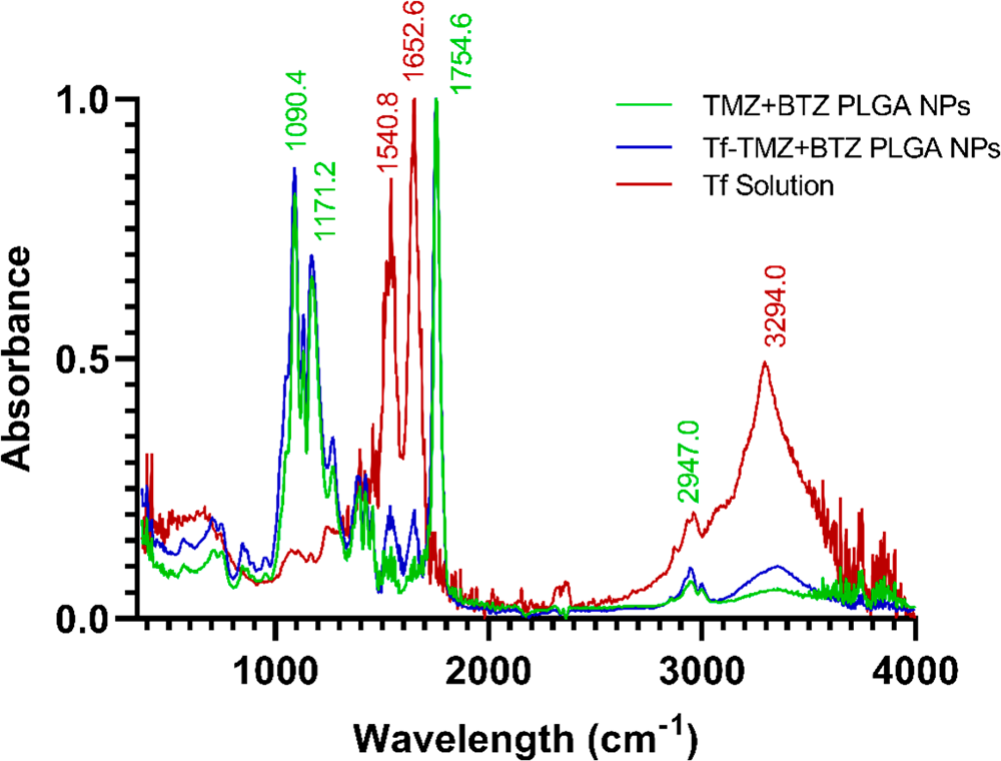
FTIR absorbance spectrum of Tf-TMZ+BTZ PLGA NPs, TMZ+BTZ PLGA NPs, and Tf stock solution recorded from 350 to 4000 cm^–1^. The peaks of PLGA are identified as green, and the peaks from Tf are identified as red.

The Tf conjugation efficiency (CE) of the prepared NPs was evaluated by using the BCA kit to quantify the unbounded Tf. The attained CE for the NPs was 70 ± 9%, corresponding to about 121 ± 10 molecules of Tf per NP. FTIR analysis was conducted to confirm the Tf conjugation further, and the results are shown in Figure 2.

Both the FTIR spectra of nonmodified and Tf-modified NPs showed the PLGA characteristic bands, corresponding to the C–O stretch (1089–1186 cm^–1^), the C–H bands (850–1450 cm^–1^), the carbonyl C=O stretching (∼1758 cm^–1^), and C–H stretches (2885–3010 cm^–1^).^33^ Furthermore, it is possible to verify that the characteristic peaks of Tf observed in the free Tf solution spectrum can also be observed, at a lower intensity, in the Tf-modified NPs, confirming the presence of Tf molecules on the tailored NPs. The Tf bands result from amide II (∼1540 cm^–1^) and amide I (∼1650 cm^–1^) vibrations and amine N–H stretching (3300–2500 cm^–1^). In addition, the characteristic bands of PLGA and Tf presented small shifts in the Tf-modified NPs, suggesting the chemical conjugation of PLGA with Tf molecules,^34^ with peaks in free Tf shifting from 1540.8 to 1539.4 cm^–1^, from 1652.6 cm^–1^ to 1647.0 cm^–1^, 3294.0 cm^–1^ (free Tf) to 3362.0 cm^–1^ in Tf-modified NPs. In addition, the PLGA characteristic peaks also showed small shifts compared with unmodified NPs, with 1090.4 cm^–1^ shifting to 1089.0 cm^–1^, 1171.2 cm^–1^ shifting to 1168.3 cm^–1^, and 1754.6 cm^–1^ shifting to 1753.2 cm^–1^.

The Tf-modified BTZ+TMZ PLGA NPs were physicochemically characterized, and the obtained results with a comparison between the nonmodified NPs are presented in Table 1.

**Table 1:** Mean Values and Standard Deviation of the Experimental Data Obtained for the Physicochemical Characterization of Tf-Conjugated and Nonconjugated TMZ+BTZ-PLGA NPs (*n* = 3)

	TMZ+BTZ loaded PLGA NPs	
	nonmodified NPs	Tf-modified NPs
mean diameter (nm)	159 ± 6	156 ± 3
PDI	0.055 ± 0.007	0.042 ± 0.016
ζ potential (mV)	–20.5 ± 1.5	–21.5 ± 1.6
EE TMZ (%)	65.4 ± 15.8	60.1 ± 8.8
EE BTZ (%)	71.1 ± 12.2	49.8 ± 12.3
TMZ LC (%)	1.7 ± 0.1	1.6 ± 0.3
BTZ LC (%)	1.9 ± 0.3	1.3 ± 0.3

The NPs’ physicochemical characteristics (size, PDI, and ζ potential values) proved to be suitable for brain delivery. The obtained sizes were below 200 nm, intended to allow efficient transport across the BBB and the accumulation in the GBM tissue due to the dimensions of vascular pores in this microenvironment.^35^ Furthermore, the NPs exhibit a negative ζ potential due to the anionic carboxyl groups of PLGA, which is advantageous for brain delivery as some studies have reported that anionic NPs are more efficiently accumulated into the brain than neutral or cationic NPs.^36^ The PDI values below 0.1 indicate the monodispersity of the NPs’ sample.

Additionally, the developed NPs showed no aggregation or significant variations in their mean size, PDI, and ζ potential after 10 weeks at storage conditions (4 °C), suggesting that the colloidal stability is preserved within this period (Tables S10 and S11 Supporting Information). The NPs’ colloidal stability is promoted by steric stabilization due to the PVA. During NPs’ formation, the PVA adsorbs to the NPs’ surface, preventing them from aggregating and improving NP stability.^37^

The values obtained for the EE were high, suggesting an adequate drug entrapment within the NPs. The higher EE values obtained for BTZ compared with TMZ (*p* < 0.05) can be explained due to TMZ’s higher water solubility, leading to a higher drug partition to the water phase during the emulsification process.

It was also concluded that Tf-conjugation did not affect NPs’ physicochemical features, proving that these NPs retain their adequate features for brain tumor delivery (*p* > 0.05). However, it was possible to verify that the EE and LC for BTZ significantly decreased with the Tf conjugation. The EE for BTZ decreased from 71.1 ± 12.1% to 49.8 ± 12.8% (*p* < 0.05), suggesting that approximately 23% of drug molecules are lost during Tf conjugation. This can be explained due the loss of BTZ molecules adsorbed to the NP’s surface during the conjugation. However, the same effect was not verified for TMZ. Despite the TMZ EE and LC values appearing to be slightly higher after Tf-conjugation, the difference is not significant (*p* > 0.05). It may be explained due to the higher TMZ’s affinity for the water, leading to lower drug adsorption to the NPs’ surface during NP’s production. The EE and LC values for TMZ and BTZ resulted in an entrapped TMZ:BTZ ratio of 1:0.8, which was applied for all the subsequent experiments.

**Figure 3:**
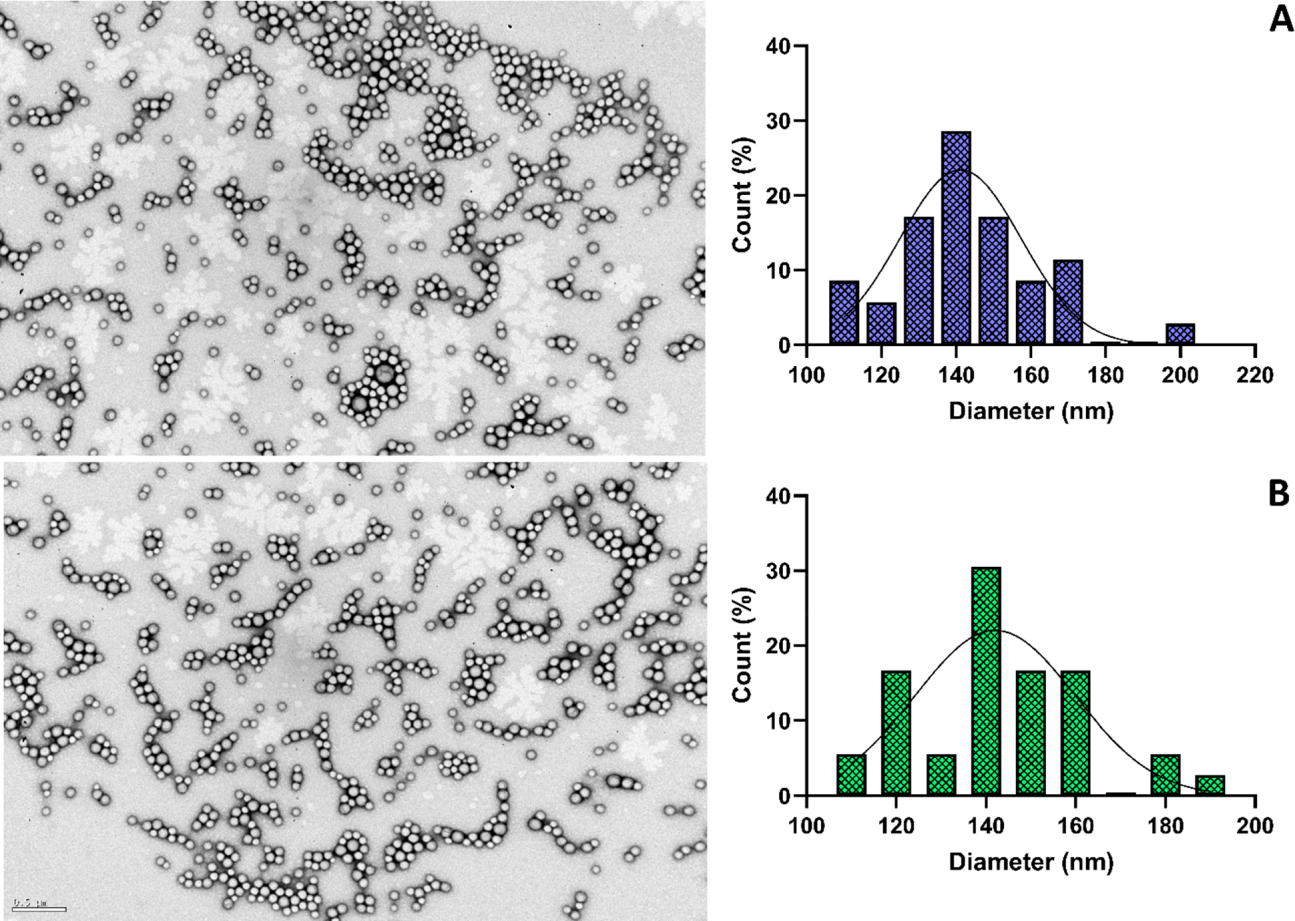
TEM images and corresponding size histograms with Gaussian distribution fitting of (A) Tf-modified and (B) nonmodified BTZ+TMZ PLGA NPs. Scale bar: 500 nm.

Furthermore, as expected, the conjugation with Tf did not influence the NPs’ storage stability (Table S11 in Supporting Information), and neither did their morphology, as observed by TEM analysis in Figure 3. Tf-modified and nonmodified NPs revealed a uniform and spherical shape with homogeneous size distribution. TEM analysis also revealed slightly smaller NPs sizes than those determined by DLS, 147 nm for nonmodified NPs and 144 nm for Tf-modified NPs, as expected due to the dispersant’s interference in the hydrodynamic diameter determination by DLS measurements.^38^ The size distribution histograms obtained from the TEM images indicated the NPs’ diameter to be within 110–200 nm and 110–190 nm for nonmodified and Tf-modified NPs, respectively, complying with the Gaussian distribution.

### 3.2 Drug Release Profile of the Synthesized NPS

**Figure 4:**
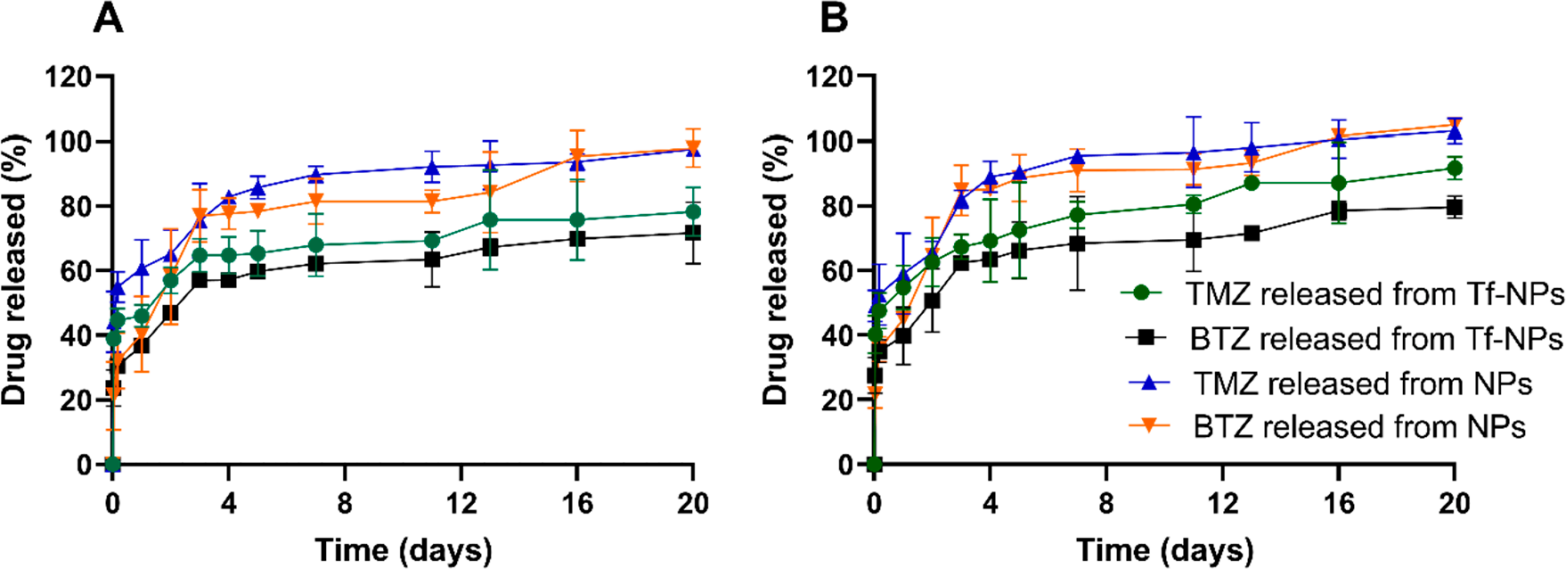
TMZ and BTZ release from BTZ+TMZ coloaded Tf-conjugated and nonconjugated PLGA NPs in different *in vitro* physiological conditions. (A) Simulated blood-circulation conditions (PBS, pH 7.4, 0.01 M at 37 °C). (B) Simulated GBM tumor acidic microenvironment (PBS, pH 6.4, 0.01 M at 37 °C). The results are shown as mean value ± SD.

The *in vitro* release of the Tf-conjugated and nonconjugated BTZ+TMZ coloaded PLGA NPs was evaluated, mimicking physiological temperature, pH, and salt concentrations of blood/healthy brain tissue (37 °C in PBS 0.01 M, NaCl 0.138 M; KCl 0.0027 M), pH 7.4) and GBM tumor microenvironment (37 °C in PBS, pH 6.4). The obtained results are represented in Figure 4.

Despite the pH of the release medium, both Tf-conjugated and nonconjugated coloaded NPs exhibited a biphasic release for both drugs. As a characteristic of PLGA NPs, it is possible to verify an initial faster release triggered by the release of the drug molecules adsorbed into the NPs’ surface. Then, a more controlled and slower release was observed for the rest of the experiment. This controlled release may occur by three different pathways: the drug diffusion from the polymeric matrix, the NPs’ surface erosion, and the bulk erosion of the polymeric matrix.^39^ In aqueous media, the polymeric surface suffers hydrolytic cleavage of its esters bonds. The acidic degradation products (lactic acid and glycolic acid) accumulate inside the polymeric matrix, thus autocatalyzing the NPs degradation and forming pores, resulting in bulk erosion. Drugs can also be released through the matrix by diffusion motivated by a concentration gradient.

Furthermore, the Tf molecules conjugated to the NPs’ surface proved to influence the release. This may be explained by the structural complexity brought by the protein, which affects drug diffusion and also may hamper the water permeation, slowing the NPs’ hydrolysis.^40^ As verified, although the NPs exhibit a similar release pattern, the release for both TMZ and BTZ is slower for the Tf-conjugated NPs than for nonmodified NPs for all the duration of the release (*p* < 0.05). For example, on the fourth day of the release 82.8 ± 0.8% of TMZ was released at pH 7.4 from nonmodified NPs, while for Tf-modified NPs only 64.7 ± 5.8% of TMZ was released (corresponding to less 18.1% TMZ released for Tf-NPs). As for BTZ at pH 7.4, the difference in the release was 20.5% (57.1 ± 0.3% for Tf-NPs vs 77.6 ± 4.8% for nonmodified NPs).

Additionally, the pH of the release buffer also affected the drug release. For example, at 24 h and acidic conditions (pH 6.4), Tf-modified NPs had released 54.8 ± 6.9% of TMZ and 39.7 ± 9.0% of BTZ, while in simulated blood circulation conditions (pH 7.4), only a release of 45.9 ± 3.4% of TMZ and 36.7 ± 0.5% of BTZ was observed (*p* < 0.05). After 4 days, while for pH 6.4 63.6 ± 0.9% of BTZ was released from Tf-modified NPs, at pH 7.4 only 57.1 ± 0.3% was released (*p* < 0.05). For nonmodified NPs, at the same time point, the acidic pH revealed more 7.6% of BTZ released than at pH 7.4 (85.2 ± 1.8% vs 77.6 ± 4.8%) (*p* < 0.05). Additionally, on the seventh day, for nonmodified NPs, 95.5 ± 1.8% of TMZ was released at pH 6.4, while at pH 7.4 only 89.7 + 2.7% was released (more 5.8%) (*p* < 0.05). The accelerated hydrolysis of the PLGA can explain this different behavior in acidic pH.

Moreover, TMZ also showed a faster release than BTZ (*p* < 0.05) in each of the pHs due to its higher affinity for the aqueous release medium, as suggested by the predicted log *P* values of −1.53 for BTZ and −0.28 for TMZ (MarvinSketch software, ChemAxon, Budapest, Hungary).

DLS measurements were performed throughout the entire experiment, and we could show that the NPs did not suffer aggregation for 20 days, both in simulated blood/healthy brain tissue and in the GBM tumor microenvironment (Tables S12–S15 in Supporting Information). Additionally, those results show a decrease in NPs’ size for both pHs, suggesting that the NPs are indeed suffering hydrolysis, corroborating the release results.

It is reported that at acidic pH, iron is released from Tf, and this iron release may result in conformational changes in the protein. As its conformation is crucial for the recognition by its receptor,^41^ FTIR and near-UV CD experiments were performed to evaluate the effect of acidic pH on the secondary and tertiary structure of the protein, respectively. The obtained results are presented in the Supporting Information (Figures S3 and S4 and Table S16).

Comparing the spectra of Tf at pH 3.8 with Tf at pH 7.4 and 6.4, the signals in the wavelength range between 270 and 290 nm appear to show small variations. The near-UV region between 270 and 290 is attributable to tyrosine residues; thus those changes may indicate alterations in the microenvironment of this amino acid.^42^ As the iron binding site includes two tyrosines, these small variations may suggest iron release at pH 3.8. However, these results are not enough to prove that changes occurred in the tertiary structure of the protein. Additionally, no major changes were verified when comparing the spectra for pH 7.4 and 6.4. These results are in agreement with previous studies reporting that iron is only released below pH 6.0,^43^ while total iron release only occurs at pH 4.^44^ Thus, iron release was not expected at pH 6.4, justifying the fact of our results not showing differences in the CD spectra between pH 7.4 and 6.4. Additionally, the FTIR results showed that iron release did not significantly change the secondary structure of the protein (*p* < 0.05), as already reported by other authors.^45^ Despite the results obtained suggesting conformation changes of the protein at pH 3.4, these are extremely acidic conditions that are not expected to occur under physiological conditions. Since at tumoral pH (6.4) no major conformational changes were observed, it is not expected that the Tf loses its biological activity.

### 3.3 NPS’ Uptake

**Figure 5:**
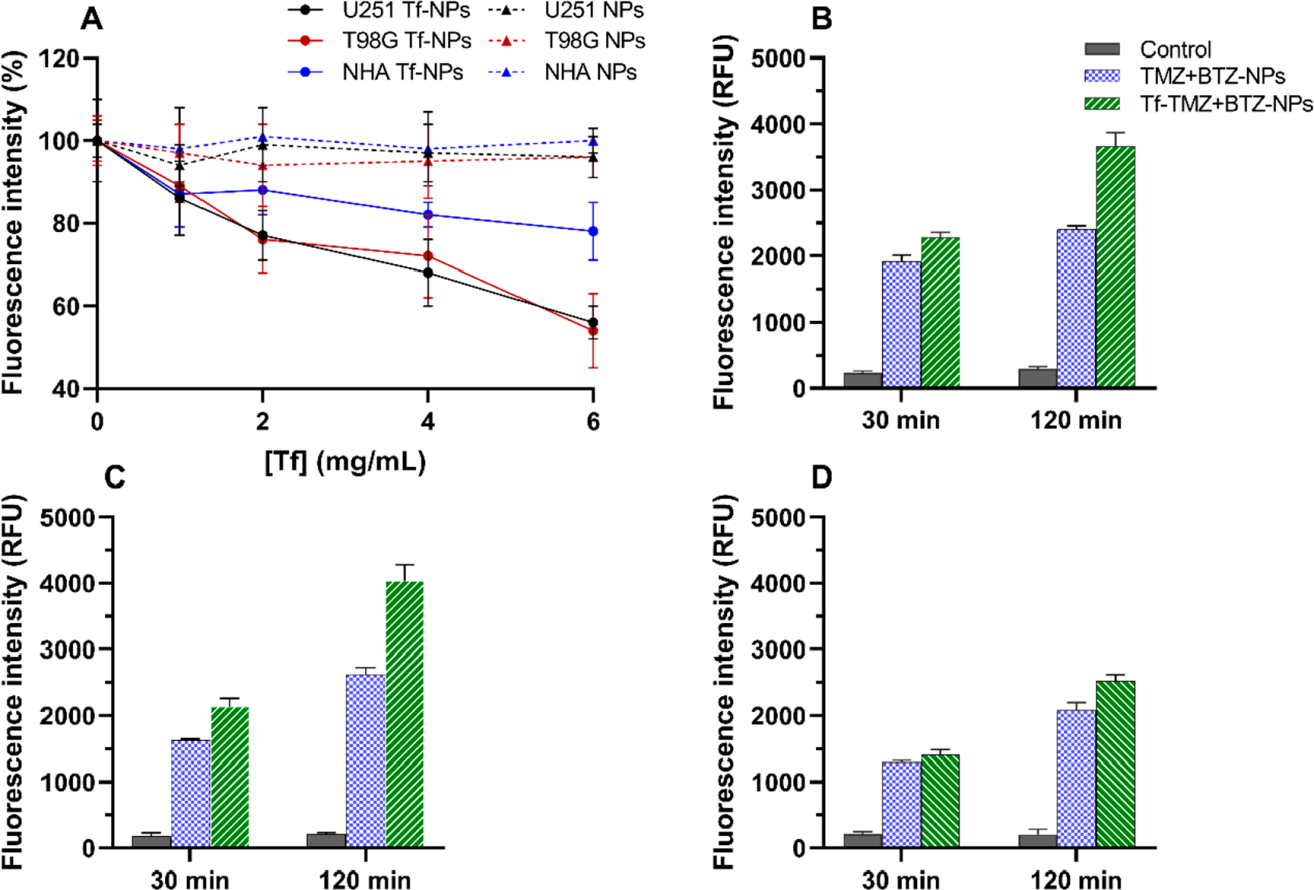
Fluorescence quantification of C6-labeled NPs uptake by human cells quantified by fluorescence. (A) U251, T98G, and NHA were incubated for 2 h with 20 μM C6-PLGA NPs cells after pretreatment with excess Tf. Evaluation of the effect of incubation period and Tf-modification of the C6-NPs uptake in (B) U251, (C) T98G, and (D) NHA cells. The cells were treated with 20 μM Tf-modified and nonmodified C6-PLGA NPs for two different incubation periods (30 and 120 min). Control refers to the autofluorescence of the nontreated cells.

The process behind the NPs uptake was investigated by competitive binding using C6-labeled NPs for fluorescence quantification. The cells were previously treated with different Tf concentrations to block the TfR and assess its impact on the internalization of Tf-conjugated and nonconjugated C6-loaded NPs. The results (Figure 5A) revealed that while pretreatment with Tf ligand did not decrease the internalization of nonconjugated NPs (*p* < 0.05), it reduced the Tf-conjugated NPs internalization in all studied cells. These results indicate that the Tf-conjugated NPs are taken up by endocytosis mediated by the TfR.

Furthermore, this uptake inhibition was more evident in tumor cells (T98G and U251 cells) than in nontumor brain cells (NHA cells). While in GBM cells, pretreatment with 6 mg·mL^–1^ of Tf led to a decrease in Tf-modified NPs uptake in about 46 ± 9% (T98G cells) and 44 ± 4% (U251 cells), in NHA cells, the observed decrease was only about 22 ± 7% (*p* < 0.05). Contrary to NHA cells, the GBM tumor cells are known to overexpress the TfR.^46^ Thus, the results showed that functionalization with Tf enhanced drug uptake more extensively in the tumor cells than in the NHA cell line. In fact, at 2 h of incubation, surface modification with Tf increased cell uptake in about 50% in both U251 and T98G cells when compared with nonmodified NPs, while in the NHA cell line Tf-NPs exhibited an increased uptake of only 20% (*p* < 0.05) (Figure 5B–D). These results are in agreement with the CD and FTIR results that show that at physiological pH no changes of the secondary and tertiary structures of Tf occur, thus not affecting its binding affinity to the TfR. The uptake studies also revealed that the chemical conjugation of PLGA carboxylic groups and Tf amine groups did not alter the protein binding ability to the TfR. Although some authors argue that targeting moieties should be conjugated to the nonbinding region of antibodies or proteins, to ensure that the antigen binding ability is not lost, several other authors showed similar results where Tf-NPs retained their binding ability after chemical conjugation through their amine group.^47,48^ It was also verified that the NPs’ internalization is time-dependent in all studied cells (*p* < 0.05).

### 3.4 Combination Index of TMZ and BTZ Drugs

**Figure 6:**
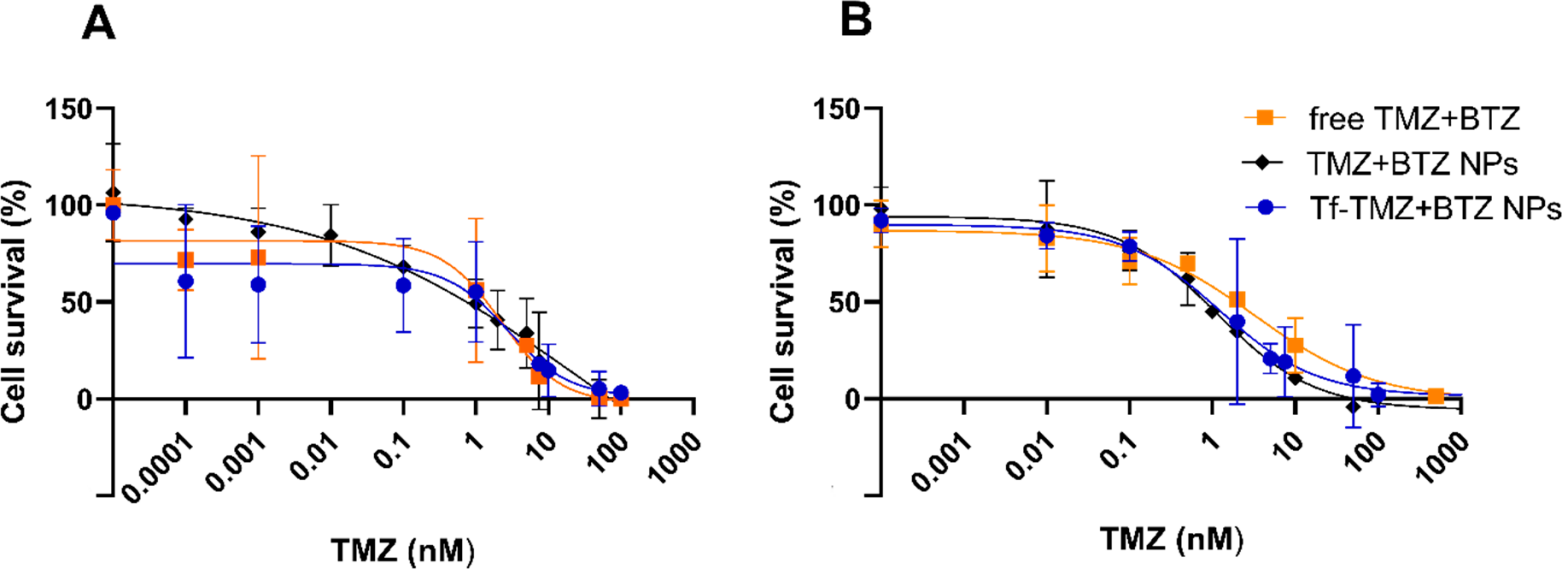
(A) U251 and (B) T98G cell survival inhibition curve after 72 h of co-treatment with TMZ and BTZ, free or loaded in nonconjugated and Tf-conjugated PLGA NPs. Data represent the mean values ± SD (*n* = 3).

Two human GBM cell lines with different MGMT expressions were used to analyze the efficacy of combined TMZ and BTZ combined therapy. Cells were treated with free BTZ, free TMZ, or free combined TMZ+BTZ at a concentration ratio of 1:0.8. This TMZ:BTZ ratio was chosen based on the EE (%) of both drugs in the NPs. The IC_50_ values were obtained from the survival inhibition curves (Figure 6 and Figure S5 in the Supporting Information) and are given in Table 2.

**Table 2:** IC_50_ Values and Combination Index and Dose Reduction Ratio (DRI) for IC_50_ Effect of TMZ+BTZ^a^

	IC_50_ (nM)			DRI	
cell line	TMZ	TMZ+BTZ	CI	TMZ	BTZ
U251	(4.1 ± 1.0) × 10^4^	26.1 ± 5.6	0.576	2.13 × 10^5^	1.74
T98G	(1.2 ± 0.9) × 10^6^	78.4 ± 10.1	0.134	6.43 × 10^6^	7.46

a The CI value indicates if the effect of the combined therapy is antagonistic (CI > 1), additive (CI = 1), or synergic (CI < 1). DRI is the dose reduction (in fold) needed to achieve the same survival inhibition when the cells are treated with each drug individually.

As well-reported in the literature,^21^ T98G cells proved to be resistant to TMZ due to *MGMT* overexpression, as shown by the higher IC_50_ values for these cells (*p* < 0.05).

Moreover, the results show that the combination of TMZ+BTZ was more efficient in inhibiting cellular proliferation than free TMZ in both cell lines, as depicted by the significantly lower IC_50_ values (*p* < 0.05) for free TMZ+BTZ (Table 2). The combination index (CI) method was further employed to evaluate if the combination of TMZ and BTZ at the chosen ratio was synergistic, additive, or antagonistic. The obtained results are presented in Table 2 (and in Table S17 and Figure S6 in the Supporting Information).

The obtained results confirmed the synergism between TMZ and BTZ at a concentration ratio of 1:0.8. As depicted by Table 2 (and Table S16 in the Supporting Information), the CI values were below 1, being lower for the resistance cells (T98G). This was expected due to the BTZ’s ability to inhibit the MGMT activity in T98G cells, leading to an increased antiproliferative activity.^8,49^ The improved anticancer activity in the MGMT-negative U251 cells can also be explained because BTZ possesses antiproliferative activity by its own (BTZ’s survival inhibition curves in Figure S7 in the Supporting Information).

Furthermore, we observed a significant reduction in the TMZ concentration required to achieve the same level of survival inhibition when combined with BTZ. This result validates the chosen concentration ratio (1:0.8) for the subsequent experiments.

### 3.5 *In Vitro* Antiproliferative Activity of the Developed NPS

The NPs’ ability to maintain the efficacy of the combined therapy after drug encapsulation was assessed using the same GBM cells. The obtained results are given in Figure 6.

The survival inhibition curves show that the combined therapy induced a concentration-dependent survival decrease in both U251 and T98G cells, regardless of whether the drugs were in their free form or entrapped in Tf-conjugated and nonconjugated NPs. The curves allowed for determination of the IC_50_ values (Table 3).

**Table 3:** IC_50_ Values at 72 h Exposure with TMZ+BTZ Loaded in TF-Conjugated and Nonconjugated TMZ+BTZ PLGA NPS^a^

	IC_50_ (nM)		
	TMZ+BTZ	TMZ+BTZ PLGA NPs	Tf-TMZ+BTZ PLGA NPs
U251	26.1 ± 5.6	5.6 ± 0.5	13.9 ± 5.2
T98G	78.4 ± 10.1	6.5 ± 1.2	8.5 ± 3.1

a Data are presented as mean values ± SD (*n* = 3).

We also verified that drug entrapment in PLGA NPs increases their efficacy in both U251 and T98G cells, as seen by the decreased IC_50_ values when compared with free drugs (*p* < 0.05). NPs enhance drug stability and uptake by the target cells, avoiding drug efflux by the P-glycoprotein. Interestingly, although both nanoformulations were more efficient than free drugs, the NPs without Tf modification appear to be more efficient in inhibiting tumor cell survival than Tf-conjugated NPs. These observations may be justified by the already mentioned slower release of the drugs from the conjugated-NPs, leading to lower effective doses of drugs.

Although both nanoformulations proved to increase the efficiency of TMZ in both MGMT-positive and MGMT-negative cells, Tf-modified NPs loaded with TMZ and BTZ are expected to be a more promising approach for GBM due to their expected ability to increase the selectivity and target delivery to the brain.

**Figure 7:**
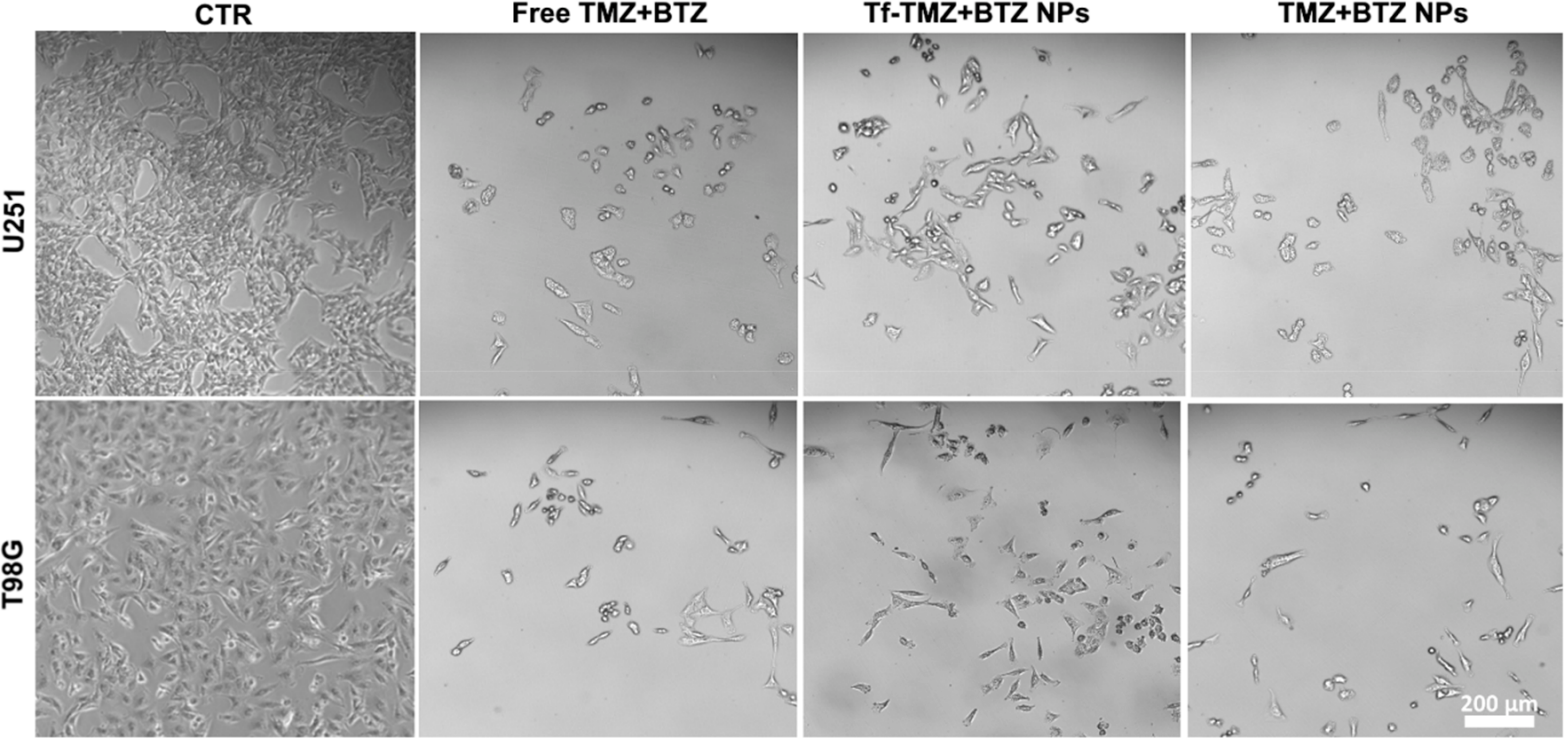
Morphological analysis of U251 and T98G cells after 72 h of treatment with combination therapy of TMZ+BTZ in the free form or entrapped in Tf-conjugated and nonconjugated PLGA NPs at IC_50_ drug dose. Control (CTR) cells were left untreated. Scale bar, 200 μm.

Furthermore, morphological analysis by fluorescence inverted microscopy revealed that the combined therapy of TMZ+BTZ induces morphological changes in treated cells, as shown in Figure 7. As expected, nontreated cells (control) exhibited high cell density and elongated shape.^50^ After treatment, the cell density significantly decreased and most cells acquired a more shrinked and spherical shape. Additionally, it is possible to observe some cells undergoing fragmentation or apoptosis and some morphological features consistent with senescence phenomena, such as flattened cells multinucleated or enlarged cell nuclei.^51^ Even though TMZ’s antiproliferative effect mainly results from apoptosis, there is evidence that TMZ can also induce cell senescence, characterized by cell cycle arrest in the G_2_-M phase triggered by the specific O^6^MeG DNA lesion.^52^ As depicted in Figure 7, the morphology and density of GBM cells were equally affected by treatment with TMZ+BTZ loaded in Tf-conjugated and nonconjugated NPs.

We also evaluated the biocompatibility of NPs in both U251 and T98G cells and in immortalized astrocytes (the NHA cell line). All cells were treated with two concentrations of control unloaded nonmodified and Tf-modified PLGA NPs (5 μM and 5 mM). The NPs were revealed to be safe, showing no significant antiproliferative effect (Figure S8 in Supporting Information) or induced morphological changes (Figure S9 in Supporting Information).

## 4 Conclusion

In this work, we proposed a targeted drug delivery strategy to knock out GBM therapy resistance mediated by the MGMT protein that severely limits the success of current therapies. PLGA NPs were designed for the co-delivery of the alkylating agent TMZ and the MGMT inhibitor BTZ. The NPs’ surface was further conjugated with Tf to increase their specificity for GBM cells.

The co-delivery of both drugs using Tf-targeting PLGA NPs efficiently sensitizes GBM cells to the antiproliferative effect of TMZ while promoting the synergistic effect of the combined therapy. This antineoplastic effect was associated with significant drug-induced cellular morphological changes due to apoptosis and senescence phenomena. Interestingly, the nonmodified NPs appeared to be more efficient in inhibiting tumor cell growth than Tf-modified NPs, due to a faster release rate that allows for an enhanced intracellular drug accumulation. Nonetheless, the developed nanoformulation may offer an innovative and effective drug delivery strategy to fight resistance mechanisms associated with the failure of current therapeutic approaches. Further studies will validate the selectivity and target delivery of Tf-modified NPs to the brain.

## Supporting Information Available

Regression equations and the statistical analysis of variance (ANOVA) of the applied regression model; physicochemical characterization of nanoparticles over time in storage conditions and simulated healthy brain/blood and brain tumor environment; TMZ and BTZ cell survival inhibition curves; combination index (CI) using the Chou–Talalay method; toxicity of bare nanoparticles (PDF)

## Abbreviations

BBB: blood–brain barrier
BCA: bicinchoninic acid
BTZ: bortezomib
C6: coumarin-6
CCD: central composite design
CE: conjugation efficiency
CI: combination index
DLS: dynamic light scattering
DMEM: Dulbecco’s modified Eagle medium
DoE: design of experiment;
EDC: ethyl-3-(3-dimethylaminopropyl) carbodiimide
EE: encapsulation efficiency
FTIR: Fourier transform infrared
GBM: glioblastoma multiforme
LC: loading capacity
MGMT: O6-methylguanine-DNA methyltransferase
NF-κB: activated B cell factor
NPs: nanoparticles
PBS: phosphate buffered saline
PDI: polydispersity index
PLGA: poly(lactic-*co*-glycolic acid)
PVA: polyvinyl alcohol
RC: regression coefficient
SRB: sulforhodamine B
TCA: trichloroacetic acid
TEM: transmission electron microscopy
Tf: transferrin
TfR: transferrin receptor
TMZ: temozolomide
