# Supplementary material for: Transferrin-Conjugated PLGA Nanoparticles for Co-Delivery of Temozolomide and Bortezomib to Glioblastoma Cells
Source: ACS Appl Nano Mater. 2023 Aug 3;6(15):14191–203. doi: 10.1021/acsanm.3c02122 (PMC10426337; doi:10.1021/acsanm.3c02122)
Supplement: Supplementary file 1 — an3c02122_si_001.pdf [file an3c02122_si_001.pdf]

Transferrin-Conjugated PLGA Nanoparticles for Co-Delivery of Temozolomide and Bortezomib to Glioblastoma Cells

Maria João Ramalho [ab †] Inês David Torres [ab †], Joana Angélica Loureiro [ab], Jorge Lima [c,d,e] and Maria Carmo Pereira [ab] **

[a] LEPABE - Laboratory for Process Engineering, Environment, Biotechnology and Energy, Faculty of Engineering, University of Porto, Rua Dr. Roberto Frias, 4200-465 Porto, Portugal

[b] ALiCE - Associate Laboratory in Chemical Engineering, Faculty of Engineering, University of Porto, Rua Dr. Roberto Frias, 4200-465 Porto, Portugal

[c] i3S - Instituto de Investigação e Inovação em Saúde, Universidade do Porto, R. Alfredo Allen, 4200-10 135 Porto, Portugal

[d] Ipatimup - Instituto de Patologia e Imunologia Molecular da Universidade do Porto, Rua Júlio Amaral de Carvalho 45, 4200-135, Porto, Portugal

[e] Faculty of Medicine of Porto University, Alameda Prof. Hernâni Monteiro, 4200-319, Porto, Portugal

* Correspondence: mjramalho@fe.up.pt; mcsp@fe.up.pt

1. PLANIFICATION OF DESIGN OF EXPERIMENT

In this work, a design of experiment (DoE) was implemented for the optimization of the NPs preparation protocol faster and more accurately by simultaneously evaluating the effect of various experimental factors while requiring a lower set of experiments.

A 2^4 full factorial design was initially implemented using the Design Expert software (11.1.2.0 version, Stat-Ease Inc., Minneapolis, USA). The chosen experimental variables were the quantity of PLGA, the PVA percentage (w/v), the sonication cycle number, and the organic solvent/water ratio, and each experimental variable was varied in a high (+1) and low level (-1), as shown in Table S1. The independent variables and experimental levels were selected based on preliminary experiments.

Table S1. Planification of the Experimental Design.

Parameters	Component	Units	Applied Level				
			-α	-1	0	+1	+α
X_1	m_{PLGA}	mg	4.5	10	20	30	35.5
X_2	PVA	%	0.09	0.5	1.25	2	2.4
X_3	sonication cycles	unit	1	2	4	6	7
X_4	O/W ratio	mL	0.43	0.5	0.625	0.75	0.82

Note: m_{PLGA} - PLGA mass; PVA – percentage of PVA; sonication cycles – number of cycles of 10 seconds each; O/W volume ratio – ratio the organic solvent and the aqueous PVA solution.

Then the factorial design was augmented by applying a Central Composite Design (CCD). For that, 2 extra levels (star points, -α and +α) were added to the model, with an α = 1.54671 (table 1). The design expert software automatically determined the α value after choosing a factorial orthogonal quadratic design augmentation.

Then, the effect of the experimental factors on each of the evaluated responses was determined by the following polynomial regression [1]:

$$Y = \beta_0 + \sum_{i=1}^{k} \beta_i X_i + \sum_{1}^{1} \beta_{ij} X_i X_j \qquad \text{Eq. 1}$$

Where Y is the predicted response; β_0 is the intercept term; $X_{i,j}$ are the independent variable levels; and $\beta_{j,i}$ are the coefficients for the levels of the variable.

The mathematical regression model was independently fitted for each response variable, and then ANOVA (Analysis of Variance) was used for the statistical analysis. p-values lower than 0.05 were considered significant at a 95% confidence interval.

2. ANALYSIS OF THE EXPERIMENTAL DESIGN

A DoE was used to optimize the TMZ-BTZ loaded PLGA NPs preparation protocol. According to preliminary experiments, four experimental variables directly affected the NPs' physicochemical characteristics. The chosen independent variables were the PLGA mass (X_1), the percentage (% w/V) of PVA (X_2), the sonication cycle number (X_3), and the ratio between the organic phase/aqueous solution (o/w) (X_4). All the remaining parameters of the preparation protocol, such as sonication amplitude, duration of sonication cycles, evaporation process, and emulsification process, were maintained constant. The NP diameter (Y_1), PDI (Y_2), zeta potential (Y_3), TMZ EE (Y_4), and BTZ EE (Y_5) were selected as the response variables.

For the initial full factorial design, 19 formulations were prepared, including 3 replicas for the center point. The DoE was then augmented to a CCD design, as the initial ANOVA statistical analysis revealed that the model was not significant for all the studied response variables (data not shown). For the design augmentation, 8 additional formulations were prepared (run 20-27). The experimental plan and the obtained results are presented in Table S2.

Table S2. Experimental plan overview and results. The coded values (-1, 0, +1 from 1 to 19 and –α, 0, +α from 20 to 27) represent the experimental levels (low, center, and high).

Run order	Coded independent variables				Response variables				
								EE (%)	
	X_1	X_2	X_3	X_4	Mean diameter (nm)	PDI	Zeta Potential (mV)	TMZ	BTZ
**1**	0	0	0	0	147.9	0.080	-17.4	60.3	67.8
**2**	-1	-1	+1	+1	192.8	0.024	-19.9	73.1	81.6
**3**	+1	+1	-1	-1	196.2	0.182	-19.1	81.0	56.0
**4**	+1	+1	+1	-1	148.1	0.041	-18.8	96.1	43.6
**5**	+1	+1	-1	+1	202.7	0.199	-21.7	71.4	51.8
**6**	-1	-1	+1	-1	194.1	0.040	-21.7	39.9	90.1
**7**	-1	+1	+1	-1	136.3	0.078	-22.5	43.9	84.7
**8**	-1	+1	+1	+1	126.7	0.064	-20.7	52.3	77.7
**9**	+1	-1	+1	-1	203.9	0.042	-23.0	92.0	76.5
**10**	-1	-1	-1	-1	174.4	0.055	-22.7	61.2	89.4
**11**	+1	+1	+1	+1	280.9	0.219	-23.1	32.8	82.0
**12**	+1	-1	+1	+1	284.7	0.178	-21.7	78.5	86.7
**13**	0	0	0	0	149.7	0.036	-19.8	66.8	95.7
**14**	-1	+1	-1	+1	142.4	0.072	-19.0	42.4	94.2
**15**	+1	-1	-1	-1	241.9	0.170	-21.4	87.6	87.7
**16**	+1	-1	-1	+1	256.8	0.186	-23.4	79.5	98.7
**17**	-1	+1	-1	-1	151.6	0.037	-17.2	53.9	73.7
**18**	0	0	0	0	155.6	0.046	-21.5	97.3	66.6
**19**	-1	-1	-1	+1	284.4	0.239	-19.4	56.5	85.1
**20**	-α	0	0	0	150.8	0.036	-24.2	36.9	52.7
**21**	+α	0	0	0	165.6	0.083	-21.7	18.8	84.7
**22**	0	-α	0	0	903.1	0.236	-22.8	52.9	80.0
**23**	0	+α	0	0	148.0	0.032	-19.7	57.9	22.2
**24**	0	0	-α	0	172.3	0.011	-15.5	95.0	41.9
**25**	0	0	+α	0	153.2	0.060	-21.2	16.0	89.4
**26**	0	0	0	-α	145.0	0.054	-24.4	46.5	70.3
**27**	0	0	0	+α	162.0	0.050	-22.7	55.7	98.3

Eq. 1 was applied to the experimental data, followed by ANOVA statistical analysis. The CCD proved to only be significant for the variables diameter (Y_1) and PDI (Y_2) ($p<0.05$) (tables S3 to S7), thus the remaining response variables (zeta potential (Y_3) TMZ EE (Y_4) BTZ EE (Y_5)) were not considered for the model.

The regression equations for diameter (Y_1), and PDI (Y_2) (Equations S1 and S2) were obtained by determining the regression coefficients (RC) describing the impact between the experimental variables and the responses (Table S8 in the Supplementary file).

2.1 EFFECT OF THE EXPERIMENTAL PARAMETERS ON THE NPS' SIZE

The NPs prepared in the CCD exhibited sizes ranging from 126.7 nm (formulation 8) to 903.1 nm (formulation 22), as shown in Table S2. In Figure S1 are represented the 2D-contour and 3D- response surface plots that provided a visual representation of the influence of the different experimental variables on the NP's diameter.

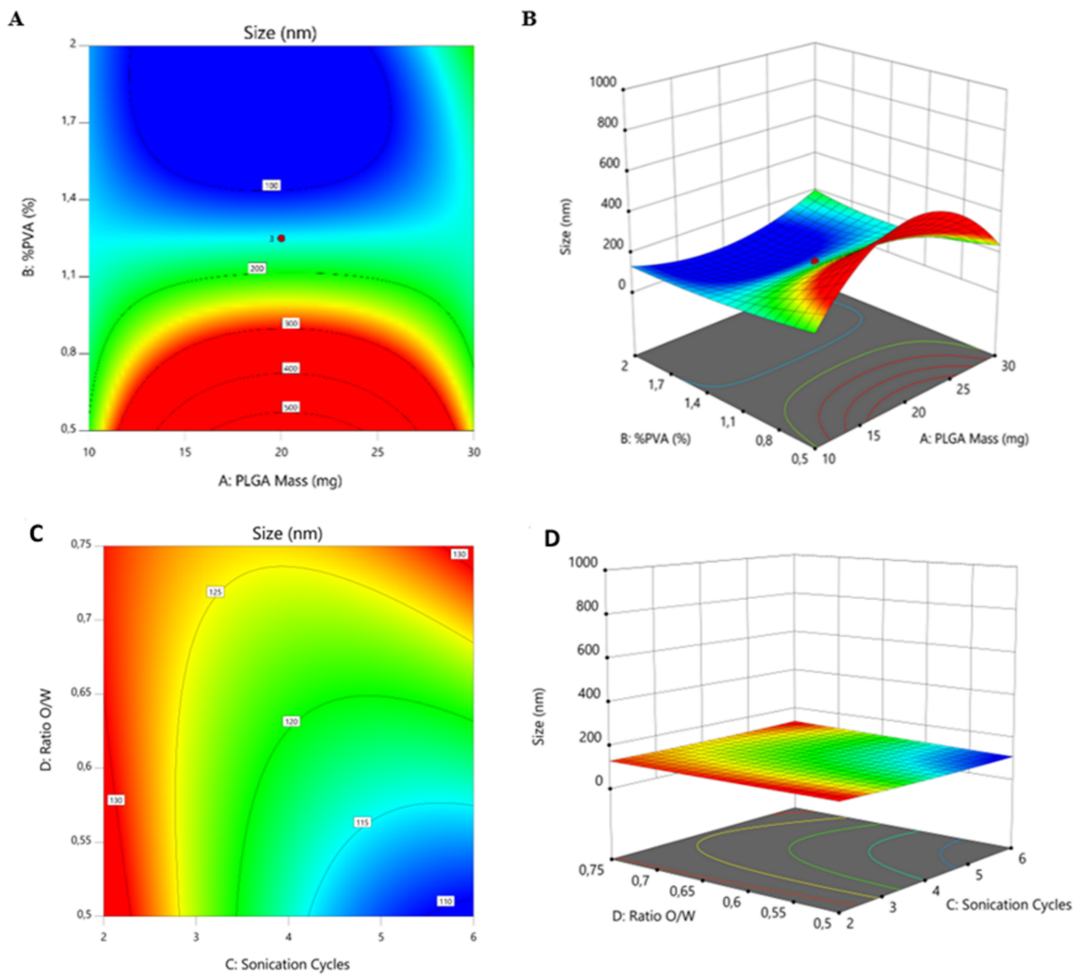

Figure S1. (A) Contour and (B) response surface plots illustrating the effect of the experimental variables, PLGA mass, and the %PVA, on the NPs' diameter. (C) Contour and (D) response surface plots illustrate the effect of the experimental variables, sonication cycles, and the O/W volume ratio on the NPs' diameter.

As shown in Figure S1 (and Table S6), the PLGA mass (X_1) and the O/W ratio (X_4) showed to influence the NPs' size positively. Decreasing the amount of PLGA leads to forming smaller NPs, since a reduced polymer concentration leads to a decreased viscosity of the organic phase. This facilitates organic solvent diffusion into the aqueous

phase, creating smaller oil droplets during emulsification [2]. The O/W ratio also increased the organic phase viscosity, positively affecting the NPs' size. On the other hand, the %PVA (X_2) and the sonication cycle number (X_3) negatively affected the NPs' size. Increasing the amount of PVA leads to smaller NPs' since PVA promotes the steric stabilization of the emulsion, decreasing the interfacial tension between the oil droplets and the continuous aqueous phase [3]. In addition, increasing the sonication cycles promotes the disruption of the emulsion droplets into ones with smaller dimensions [4].

2.2 EFFECT OF THE EXPERIMENTAL PARAMETERS ON THE NPS' PDI

The PDI of the NPs ranged from 0.011 (formulation 24) to 0.239 (formulation 19), as shown in Table S2. In Figure S2 are presented the 2D contour plots and the 3D response surface plots illustrating the effect of the experimental variables on the NPs' PDI values.

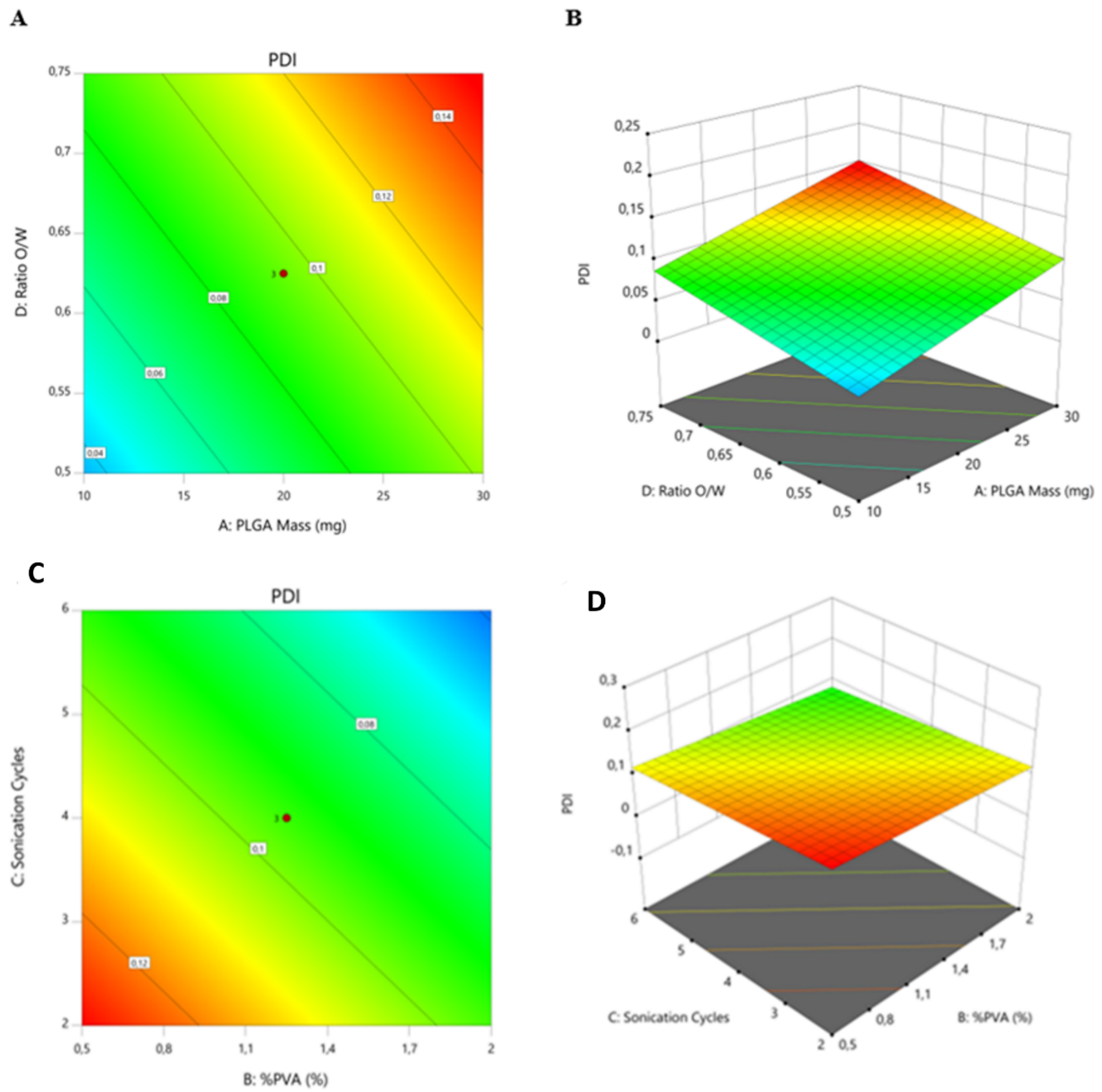

Figure S2. (A) Contour and (B) response surface plots illustrate the experimental variables' effect, PLGA mass, and the O/W volume ratio on the NPs' PDI. (C) Contour and (D) response surface plots illustrate the experimental variables' effect, the %PVA and sonication cycles, on the NPs' PDI.

As shown in Figure S2 and Table S8, the PLGA mass and the O/W ratio revealed a positive impact on the PDI value of the NPs. Similarly, as verified for NPs' diameter, decreasing the organic phase's viscosity facilitates the organic solvent diffusion into the aqueous phase and the generation of a more homogeneous sample. On the other hand, the PVA amount and sonication cycle number negatively affected the response variable, increasing the homogeneity of the NPs' suspension.

Table S3. ANOVA analysis for the response variable diameter (Y_1) applying a quartic model.

Diameter (Y_1)						
**Source**	**Sum of Squares**	**df**	**Mean Square**	**F-value**	**p-value**	
**Model**	5.569×10^5	24	23205.54	1430.38	0.0007	significant
X_1-PLGA Mass	109.52	1	109.52	6.75	0.1217	
X_2-%PVA	2.851×10^5	1	2.851×10^5	17572.71	< 0.0001	
X_3-Sonication Cycles	182.41	1	182.41	11.24	0.0786	
X_4-Ratio O/W	144.50	1	144.50	8.91	0.0963	
X_1X_2	1044.91	1	1044.91	64.41	0.0152	
X_1X_3	944.03	1	944.03	58.19	0.0168	
X_1X_4	1315.88	1	1315.88	81.11	0.0121	
$X_2 X_3$	411.08	1	411.08	25.34	0.0373	
$X_2 X_4$	439.95	1	439.95	27.12	0.0350	
$X_3 X_4$	405.02	1	405.02	24.97	0.0378	
X_1^2	61.06	1	61.06	3.76	0.1919	
X_2^2	1.683×10^5	1	1.683×10^5	10373.04	< 0.0001	
X_3^2	163.80	1	163.80	10.10	0.0864	
X_4^2	7.11	1	7.11	0.4380	0.5762	
**Pure Error**	32.45	2	16.22			
**Cor Total**	5.570×10^5	26				

Table S4. ANOVA analysis for the response variable PDI (Y_2) applying a linear model.

PDI (Y_2)						
**Source**	**Sum of Squares**	**df**	**Mean Square**	**F-value**	**p-value**	
**Model**	0.0488	4	0.0122	2.90	0.0454	significant
X_1-PLGA Mass	0.0223	1	0.0223	5.30	0.0312	
X_2-%PVA	0.0062	1	0.0062	1.46	0.2395	
X_3-Sonication Cycles	0.0069	1	0.0069	1.64	0.2142	
X_4-Ratio O/W	0.0135	1	0.0135	3.21	0.0870	
**Residual**	0.0926	22	0.0042			
Lack of Fit	0.0915	20	0.0046	8.60	0.1092	not significant
Pure Error	0.0011	2	0.0005			
**Cor Total**	0.1414	26				

Table S5. ANOVA analysis for the response variable Zeta Potential (Y_3) applying a quadratic model.

Zeta Potential (Y_3)						
**Source**	**Sum of Squares**	**df**	**Mean Square**	**F-value**	**p-value**	
**Model**	86.85	14	6.20	1.87	0.1424	not significant
X_1-PLGA Mass	1.32	1	1.32	0.3966	0.5407	
X_2-%PVA	12.16	1	12.16	3.66	0.0799	
X_3-Sonication Cycles	12.81	1	12.81	3.86	0.0732	
X_4-Ratio O/W	0.0008	1	0.0008	0.0002	0.9878	
X_1X_2	0.3906	1	0.3906	0.1176	0.7376	
X_1X_3	1.89	1	1.89	0.5690	0.4652	
X_1X_4	10.08	1	10.08	3.03	0.1071	
$X_2 X_3$	4.73	1	4.73	1.42	0.2558	
$X_2 X_4$	7.98	1	7.98	2.40	0.1471	
$X_3 X_4$	0.8556	1	0.8556	0.2575	0.6210	
X_1^2	7.47	1	7.47	2.25	0.1596	
X_2^2	0.1080	1	0.1080	0.0325	0.8599	
X_3^2	14.23	1	14.23	4.28	0.0607	
X_4^2	12.83	1	12.83	3.86	0.0730	
**Residual**	39.87	12	3.32			
Lack of Fit	31.38	10	3.14	0.7396	0.6978	not significant
Pure Error	8.49	2	4.24			
**Cor Total**	126.72	26				

Table S6. ANOVA analysis for the response variable EE TMZ (Y_4) applying a quadratic model.

EE TMZ (Y_4)						
Source	Sum of Squares	df	Mean Square	F-value	p-value	
**Model**	5348.59	14	382.04	0.5667	0.8453	not significant
X_1-PLGA Mass	1350.05	1	1350.05	2.00	0.1825	
X_2-%PVA	364.66	1	364.66	0.5409	0.4762	
X_3-Sonication Cycles	1043.29	1	1043.29	1.55	0.2372	
X_4-Ratio O/W	144.59	1	144.59	0.2145	0.6516	
X_1X_2	20.25	1	20.25	0.0300	0.8653	
X_1X_3	14.21	1	14.21	0.0211	0.8870	
X_1X_4	897.90	1	897.90	1.33	0.2709	
$X_2 X_3$	31.19	1	31.19	0.0463	0.8333	
$X_2 X_4$	429.73	1	429.73	0.6375	0.4401	
$X_3 X_4$	0.0900	1	0.0900	0.0001	0.9910	
X_1^2	737.83	1	737.83	1.09	0.3161	
X_2^2	138.87	1	138.87	0.2060	0.6580	
X_3^2	143.07	1	143.07	0.2122	0.6533	
X_4^2	32.85	1	32.85	0.0487	0.8290	
**Residual**	8089.67	12	674.14			
Lack of Fit	7306.73	10	730.67	1.87	0.3989	not significant
Pure Error	782.94	2	391.47			
**Cor Total**	13438.26	26				

Table S7. ANOVA analysis for the response variable EE BTZ (Y_5) applying a quadratic model.

EE BTZ (Y_5)						
Source	Sum of Squares	df	Mean Square	F-value	p-value	
**Model**	5371.72	14	383.69	1.07	0.4581	not significant
X_1-PLGA Mass	93.43	1	93.43	0.2606	0.6190	
X_2-%PVA	2360.39	1	2360.39	6.58	0.0247	
X_3-Sonication Cycles	172.33	1	172.33	0.4807	0.5013	
X_4-Ratio O/W	476.20	1	476.20	1.33	0.2715	
X_1X_2	627.88	1	627.88	1.75	0.2104	
X_1X_3	0.5366	1	0.5366	0.0015	0.9698	
X_1X_4	187.21	1	187.21	0.5222	0.4837	
$X_2 X_3$	91.92	1	91.92	0.2564	0.6218	
$X_2 X_4$	97.17	1	97.17	0.2711	0.6121	
$X_3 X_4$	6.64	1	6.64	0.0185	0.8940	
X_1^2	60.01	1	60.01	0.1674	0.6896	
X_2^2	296.08	1	296.08	0.8259	0.3814	
X_3^2	11.69	1	11.69	0.0326	0.8597	
X_4^2	890.24	1	890.24	2.48	0.1410	
**Residual**	4301.94	12	358.49			
Lack of Fit	3762.40	10	376.24	1.39	0.4883	not significant
Pure Error	539.54	2	269.77			
**Cor Total**	9673.66	26				

Table S8. Regression coefficients (RC) and p-values for the significant response variables Y_1 and Y_2. The positive sign before the coefficient indicates a positive effect with increased response, while a negative sign indicates a decreased response.

	**Diameter – Y_1**		**PDI – Y_2**	
	**RC**	**p-Value**	**RC**	**p-Value**
**X_1-PLGA Mass**	4.78	0.1217	0.0327	0.0312
**X_2-%PVA**	-244.10	< 0.0001	-0.0172	0.2395
**X_3-Sonication Cycles**	-6.17	0.0786	-0.0182	0.2142
**X_4-Ratio O/W**	5.50	0.0963	0.0255	0.0870
**X_1X_2**	8.08	0.0152		
**X_1X_3**	7.68	0.0168		
**X_1X_4**	9.07	0.0121		
**X_2 X_3**	5.07	0.0373		
**X_2 X_4**	-5.24	0.0350		
**X_3 X_4**	5.03	0.0378		

Equation S1: Diameter = 151.07 + 4.78 (PLGA mass) – 244.10 (%PVA) – 6.17 (Sonication Cycles)

+ 5.50 (O/W Ratio) + 8.08 (PLGA mass) (%PVA)

+ 7.68 (PLGA mass) (Sonication Cycles)

+ 9.07 (PLGA mass) (O/W Ratio)

+ 5.07 (%PVA) (Sonication Cycles)

- 5.24 (%PVA) (O/W Ratio)

+ 5.03 (Sonication Cycles) (O/W Ratio)

Equation S2: PDI = 0.0944 + 0.0327 (PLGA mass) – 0.0172 (%PVA) – 0.0182 (Sonication Cycles) + 0.0255 (O/W Ratio)

2.3 PROTOCOL OPTIMIZATION AND MODEL VALIDATION

Optimized NPs were produced after the validation of the experimental design by determining the optimal formulation parameters from the regression equations. For that, range limits were established the for each response. The NPs' diameter range was set

between 150-180 nm (Y1); for the PDI, the range was set at 0.011-0.1 (Y_2). The determined optimal levels of the independent variables were 19 mg of PLGA, 1.26% (w/v) of PVA, 4 sonication cycles, and a 0.667 O/W ratio. A formulation checkpoint was prepared in triplicate to validate the protocol, applying those optimal experimental values. In table S9 are presented the predicted responses and the obtained experimental results.

Table S9. Validation model; Comparison between the predicted and the experimental values. The experimental results are represented as the mean value (n=3) and the range of the obtained experimental values.

	**Predicted Response**	**Experimental Values**
**Diameter (nm)**	149<br>(135-163)	159<br>(152-164)
**PDI**	0.099<br>(0.017-0.182)	0.055<br>(0.048-0.062)
**Zeta Potential (mV)**	n.a.	-20.5<br>(-19.1-[-22.0])
**TMZ EE (%)**	n.a.	65.4<br>(50.2-86.5)
**BTZ EE (%)**	n.a.	71.1<br>(58.0-87.2)

As observed in Table S9, both diameter and PDI responses of the checkpoint formulations were within the predicted range, validating the mathematical model. Although excluded from the model, the zeta potential and the encapsulation efficiency values were also evaluated.

3. EVALUATION OF NPS' STABILITY

Table S10. Mean size, PDI and zeta potential values for non-conjugated TMZ+BTZ loaded PLGA NPs in storage conditions (aqueous suspension in ultrapure water, 4 ºC). Data are represented as mean ± SD (n=3).

	**Diameter (nm)**	**PDI**	**Zeta Potential (mV)**
Week 1	159 ± 6	0.055 ± 0.007	-20.5 ± 1.5
Week 3	155 ± 3	0.093 ± 0.020	-21.4 ± 0.8
Week 5	151 ± 0	0.059 ± 0.003	-20.2 ± 1.6
Week 6	150 ± 2	0.288 ± 0.371	-21.0 ± 0.4
Week 7	146 ± 1	0.056 ± 0.002	-17.6 ± 3.5
Week 8	149 ± 2	0.061 ± 0.015	-23.2 ± 1.7
Week 10	145 ± 1	0.066 ± 0.011	-19.0 ± 1.6

Table S11. Mean size, PDI and zeta potential values for Tf conjugated TMZ+BTZ loaded PLGA NPs in storage conditions (aqueous suspension in ultrapure water, 4 ºC). Data are represented as mean ± SD (n=3).

	**Diameter (nm)**	**PDI**	**Zeta Potential (mV)**
Week 1	156 ± 3	0.042 ± 0.0016	-21.5 ± 1.6
Week 3	158 ± 12	0.037 ± 0.019	-20.3 ± 1.9
Week 5	153 ± 5	0.047 ± 0.009	-21.6 ± 1.9
Week 6	152 ± 8	0.041 ± 0.009	-22.2 ± 2.4
Week 7	161 ± 3	0.072 ± 0.005	-18.5 ± 1.4
Week 8	149 ± 8	0.055 ± 0.030	-20.5 ± 1.9
Week 10	145 ± 13	0.040 ± 0.030	-18.4 ± 1.6

Table S12. Mean size, PDI and zeta potential values for non-conjugated TMZ+BTZ loaded PLGA NPs in simulate blood conditions (37 ºC, PBS, pH 7.4). Data are presented as mean ± SD (n=3). A decreased in zeta potential values when compared with NPs in ultrapure water was verified due to the free charges of PBS that interact with the PLGA surface carboxylic groups. However, despite these lower zeta potential values, the NPs did not aggregate. A decrease in NPs size was observed, suggesting that the NPs undergo hydrolysis in the release buffer.

	**Diameter (nm)**	**PDI**	**Zeta Potential (mV)**
Day 0	159 ± 6	0.055 ± 0.007	-7.8 ± 1.3
Day 8	142 ± 8	0.061 ± 0.023	-11.1 ± 2.1
Day 12	145 ± 6	0.080 ± 0.027	-9.7 ± 3.2
Day 16	144 ± 1	0.080 ± 0.050	-9.5 ± 2.1
Day 20	139 ± 2	0.073 ± 0.029	-12.3 ± 3.9

Table S13. Mean size, PDI and zeta potential values for Tf conjugated TMZ+BTZ loaded PLGA NPs in simulate blood conditions (37 ºC, PBS, pH 7.4). Data are presented as mean ± SD (n=3). A decreased in zeta potential values when compared with NPs in ultrapure water was verified due to the free charges of PBS that interact with the PLGA surface carboxylic groups. However, despite these lower zeta potential values, the NPs did not aggregate. A decrease in NPs size was observed, suggesting that the NPs undergo hydrolysis in the release buffer.

	**Diameter (nm)**	**PDI**	**Zeta Potential (mV)**
Day 0	156 ± 3	0.042 ± 0.0016	-6.4 ± 0.2
Day 8	142 ± 3	0.086 ± 0.031	-12.4 ± 4.0
Day 12	145 ± 6	0.068 ± 0.037	-15.1 ± 4.0
Day 16	144 ± 3	0.086 ± 0.005	-8.7 ± 4.7
Day 20	138 ± 1	0.097 ± 0.027	-9.7 ± 4.0

Table S14. Mean size, PDI and zeta potential values for non-conjugated TMZ+BTZ loaded PLGA NPs in simulated tumor acidic environment (37 ºC, PBS, pH 6.4). Data are represented as mean ± SD (n=3). In acidic environment, the carboxylic groups of the polymer are protonated leading to almost neural zeta potential values. However, the NPs did not aggregate during the studied period. A decrease in NPs size was observed, suggesting that the NPs undergo hydrolysis in the release buffer.

	**Diameter (nm)**	**PDI**	**Zeta Potential (mV)**
Day 0	159 ± 6	0.055 ± 0.007	-1.6 ± 0.2
Day 8	149 ± 2	0.082 ± 0.010	-2.3 ± 0.8
Day 12	144 ± 4	0.084 ± 0.039	-1.7 ± 0.7
Day 16	151 ± 3	0.119 ± 0.009	-2.7 ± 0.4
Day 20	140 ± 6	0.085 ± 0.015	-2.6 ± 0.8

Table S15. Mean size, PDI and zeta potential values for Tf conjugated TMZ+BTZ loaded PLGA NPs in in simulated tumor acidic environment (37 ºC, PBS, pH 6.4). Data is represented as mean ± SD (n=3). In acidic environment, the carboxylic groups of the polymer are protonated leading to almost neural zeta potential values. However, the NPs did not aggregate during the studied period. A decrease in NPs size was observed, suggesting that the NPs undergo hydrolysis in the release buffer.

	**Diameter (nm)**	**PDI**	**Zeta Potential (mV)**
Day 0	156 ± 3	0.042 ± 0.0016	-2.3 ± 0.4
Day 8	141 ± 2	0.157 ± 0.042	-2.3 ± 2.1
Day 12	137 ± 3	0.078 ± 0.048	-1.6 ± 0.4
Day 16	157 ± 2	0.084 ± 0.012	-2.7 ± 1.2
Day 20	149 ± 11	0.090 ± 0.072	-3.3 ± 0.9

4. CD EXPERIMENTS FOR TF TERTIARY STRUCTURE EVALUATION

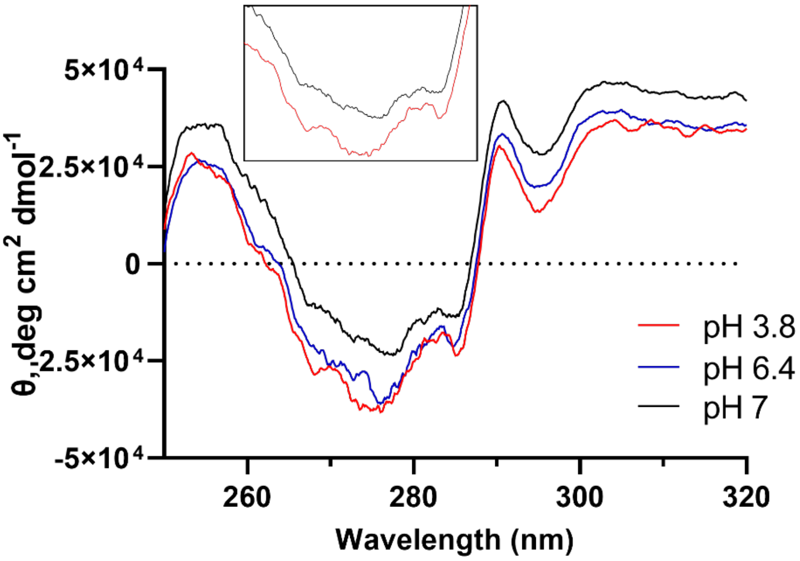

Figure S3. Near UV-CD spectra of Tf at different pH (7.4, 6.4 and 3.8). Inset shows the region between 270-290 observed for pH 7.4 and 3.8. The CD spectrum in the near UV region (250-320 nm) reflects the environments of the aromatic amino acid side chains, thus providing information about proteins' tertiary structure. Signals at wavelength range 250–270 nm are attributed to phenylalanine residues, those at are attributed 270–290 nm to tyrosine, and those at 290–300 nm to tryptophan.

5. FTIR EXPERIMENTS FOR TF SECONDARY STRUCTURE EVALUATION

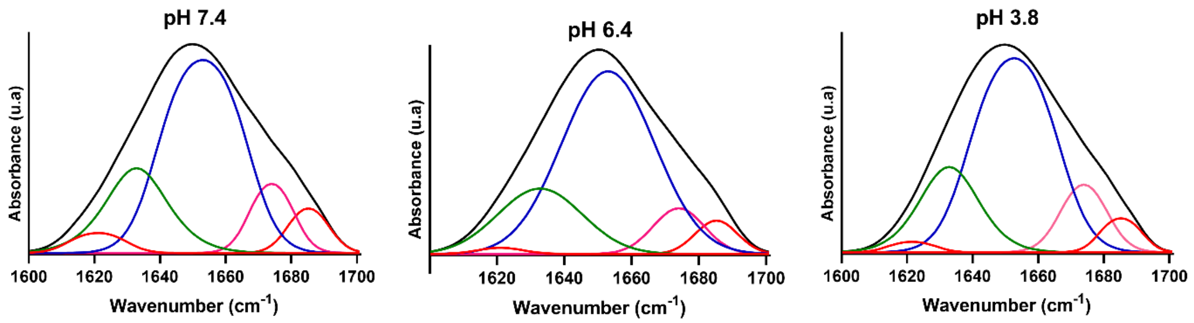

Figure S4. Deconvolution of FTIR spectra of Tf at different pH (7.4, 6.4 and 3.8) from 1600 to 1700 nm using the Gaussian-Lorentzian function. Antiparallel β-sheet structures exhibit two typical bands, 1615–1627 cm^{-1} and 1674–1695 cm^{-1}. Parallel β-sheet, β-turn and α-helix structures display bands around 1623–1641 cm^{-1}, 1662–1686 cm^{-1} and 1648–1657 cm^{-1}, respectively. Antiparallel β-sheet, parallel β-sheet, α-helix, and β-turn structures are presented in red, green, blue and pink lines, respectively.

Table S16. Quantification of Tf's secondary structures at different pH. The values were obtained from the Gaussian-Lorentzian deconvolution of the FTIR spectra and are presented as mean ± SD (n=3). No statistical difference was observed between groups. The obtained results are in agreement with previous studies reporting that iron release does not affect the protein secondary structure, and that both apo and holo-forms of the protein possess about 53% of α-helix, 23% of parallel β-sheet and 10% of β-turn structures.

	**pH 7.4**	**pH 6.4**	**pH 3.8**
**Antiparallel β-sheet**	10.1 ± 3.3	4.3 ± 3.0	5.0 ± 2.3
**Parallel β-sheet**	22.9 ± 8.1	21.6 ± 1.2	21.9 ± 1.2
**α-helix**	56.5 ± 7.0	62.1 ± 2.6	61.2 ± 1.7
**β-turn**	10.5 ± 5.3	9.7 ± 1.3	11.9 ± 3.1

6. CELL EXPERIMENTS

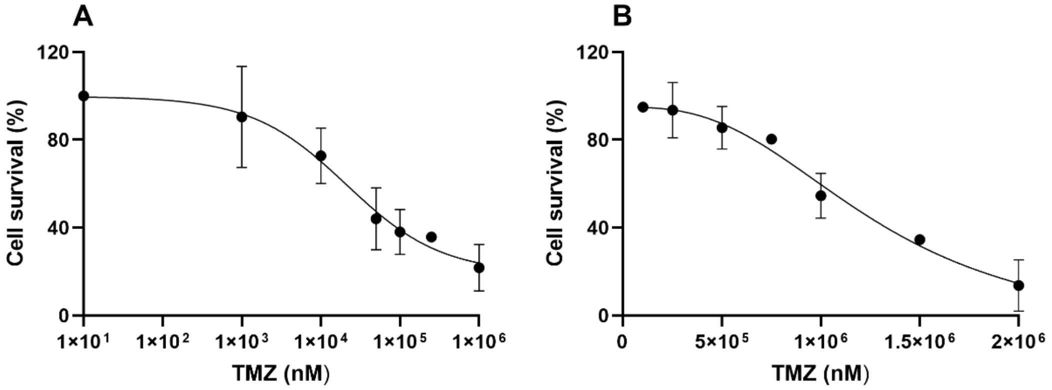

Figure S5. Cell survival inhibition curve after 72 h treatment with free TMZ, two GBM human cells by SRB assay. (A) U251 cells and (B) T98G cells. Cell survival is presented as percent [(%) = ((T)/(C)) x 100]. Data represented as mean ± SD (n=3). IC50 values of 4.1 ± 1.0 x 10^4 nM for U251 cells and 1.2 ± 0.9 x 10^6 nM for T98G line were obtained from the regression of the curves.

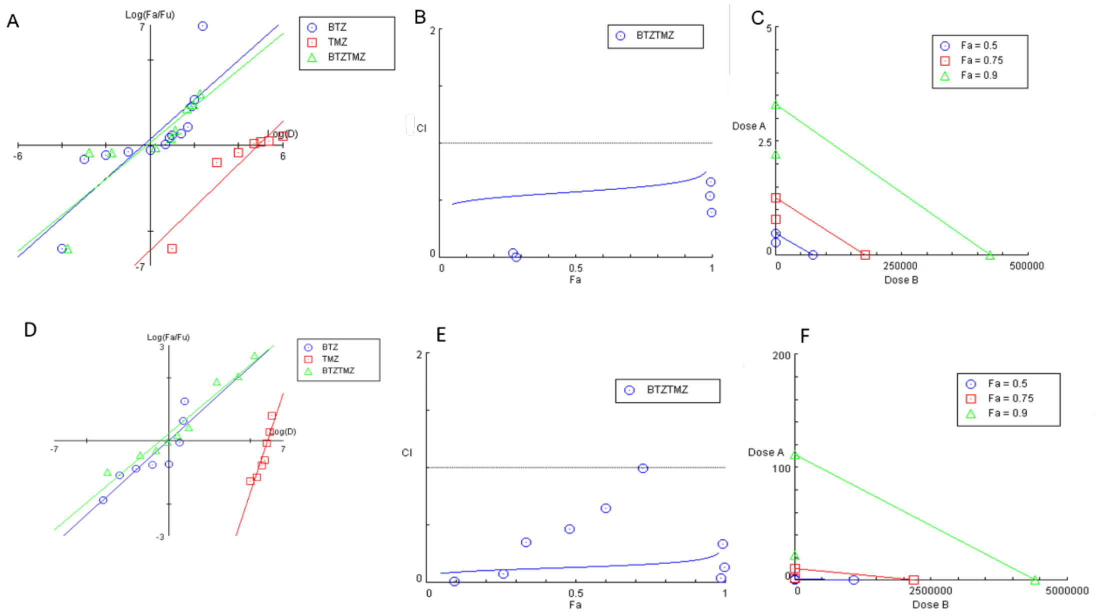

Figure S6. Median-effect plot for (A) U251 cells and (D) T98G cells. Combination index plot for (B) U251 cells and (E) T98G cells. The CI value gives an indication if the effect of the combined therapy is synergic (CI < 1), additive (CI =1) or antagonistic (CI > 1). For U251 cells, all combination data points are located in the synergy side (CI < 1). For T98G cells, only one point fall in the additive line (CI=1), and the remaining indicate synergy. Isobolograms for 50 % (Fa 0.5), 75 % (Fa 0.75), and 90 % (Fa 0.9) inhibition for (C) U251 cells and (F) T98G cells. If the data points are located on the diagonal line this indicates an additive effect of the combined therapy. If the data points fall on the lower left or on the upper right of the diagonal line, it indicates synergistic or antagonistic effect, respectively. For both U251 and T98G cells, all three studied effects (IC_{50}, IC_{75} and IC_{90}) revealed synergism.

Table S17. Combination Index and Dose Reduction Ratio (DRI) of TMZ+BTZ. DRI is the dose reduction (in fold) needed to achieve the same survival inhibition when the cells are treated with each drug individually.

Cell line	Effect	CI	DRI	
			TMZ	BTZ
**U251**	IC50	0.576	2.13×10^5	1.74
	IC75	0.622	1.81×10^5	1.61
	IC90	0.672	1.54×10^5	1.49
	IC95	0.708	1.37×10^5	1.41
**T98G**	IC50	0.134	6.43×10^6	7.46
	IC75	0.162	1.02×10^6	6.16
	IC90	0.196	1.61×10^5	5.09
	IC95	0.224	4.61×10^4	4.47

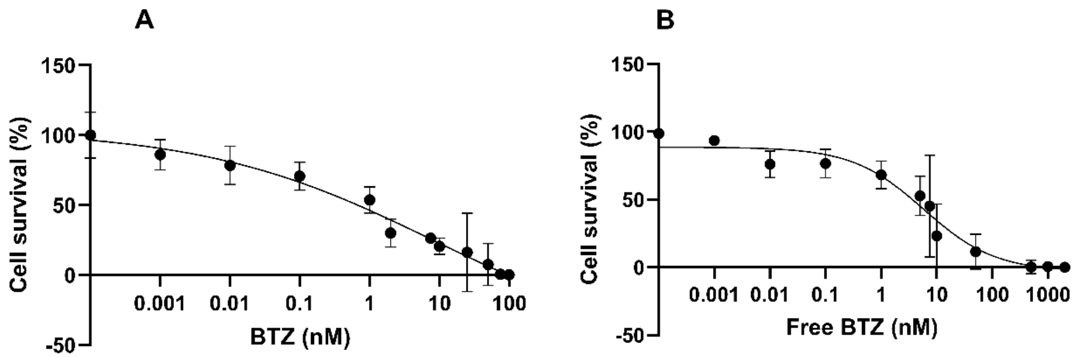

Figure S7. Cell survival inhibition curve after 72 h treatment with free BTZ, two GBM human cells by SRB assay. (A) U251 cells and (B) T98G cells. Cell survival is presented as percent [(%) = ((T)/(C)) x 100]. Data represented as mean ± SD (n=3).

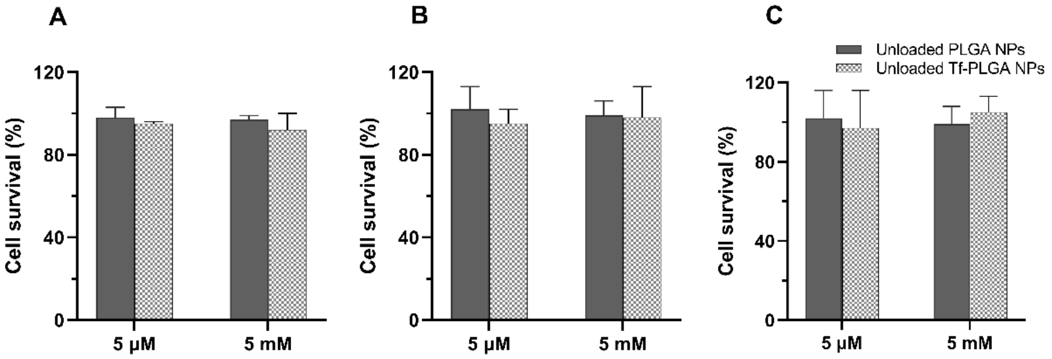

Figure S8. Cell survival of (A) U251, (B) T98G and (C) NHA cells after 72 h treatment with 5 μM and 5 mM of unloaded non-modified and Tf-modified PLGA NPs. The concentrations were chosen based to test the working concentration of drug-loaded NPs (5 μM) and excess concentration (5 mM). Cell survival is presented as percent [(%) = ((T)/(C)) x 100]. Data represented as mean ± SD (n=3). No significant cytotoxic effect was observed the two concentrations in all studied human cell lines ($p>0.05$).

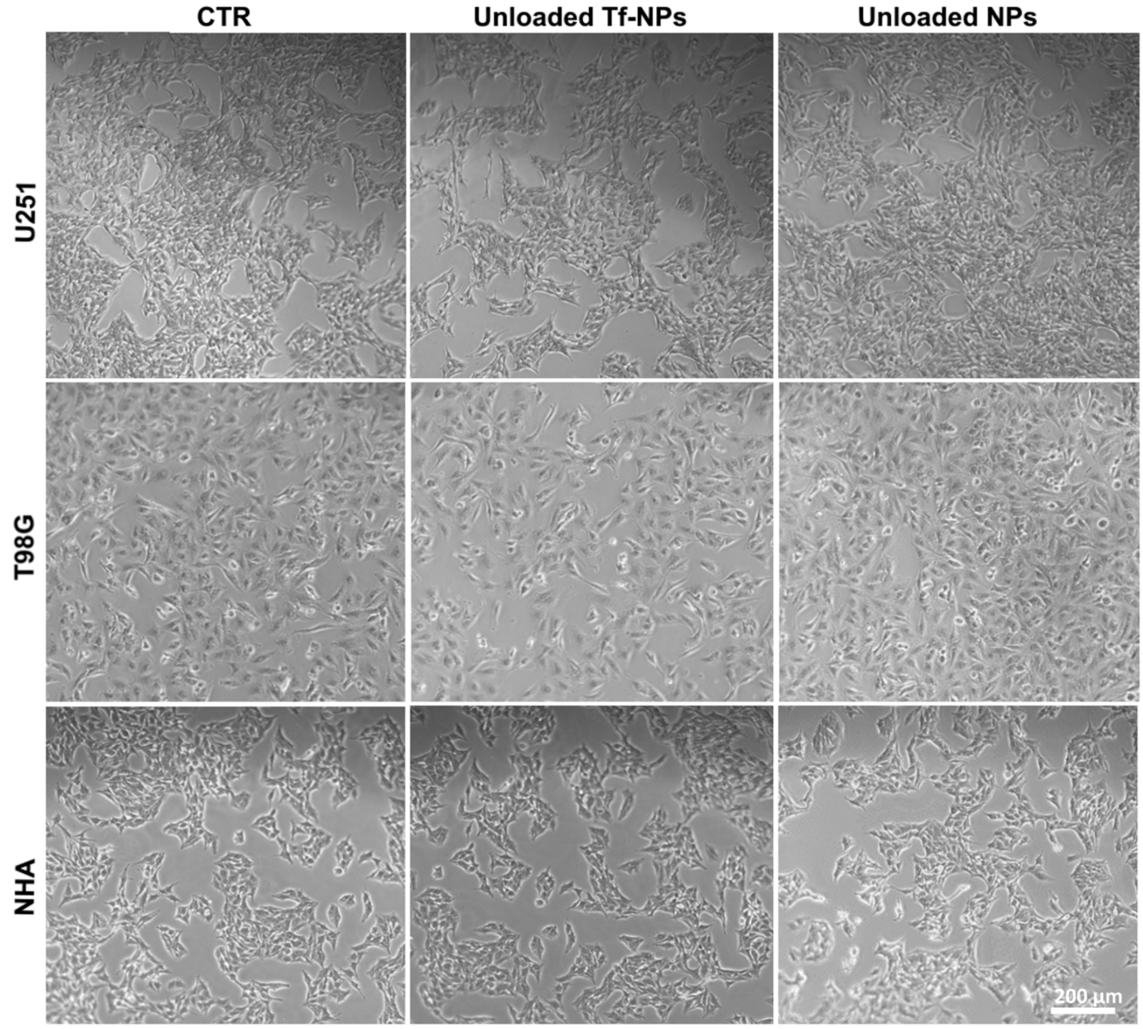

Figure S9. Morphological analysis of U251, T98G and NHA cells after 72 hours treatment with 5mM of unloaded Tf-modified and non-modified PLGA NPs. The concentrations were chosen based to test the working concentration of drug-loaded NPs (5 μM) and excess concentration (5 mM). Control (CTR) cells were left untreated. Scale bar 200 μm. No major morphological changes were observed in all studied human cell lines.

REFERENCES

1. Cun, D.; Jensen, D. K.; Maltesen, M. J.; Bunker, M.; Whiteside, P.; Scurr, D.; Foged, C.; Nielsen, H. M. High loading efficiency and sustained release of siRNA encapsulated in PLGA nanoparticles: quality by design optimization and characterization. *European journal of pharmaceutics and biopharmaceutics* **2011,** *77* (1),26-35. https://doi.org/10.1016/j.ejpb.2010.11.008

2. Ramalho, M. J.; Loureiro, J. A.; Coelho, M. A.; Pereira, M. C. Factorial Design as a Tool for the Optimization of PLGA Nanoparticles for the Co-Delivery of Temozolomide and O6-Benzylguanine. *Pharmaceutics* **2019,** *11* (8),401. https://doi.org/10.3390/pharmaceutics11080401

3. Liu, Y.; Pan, J.; Feng, S.-S. Nanoparticles of lipid monolayer shell and biodegradable polymer core for controlled release of paclitaxel: effects of surfactants on particles size, characteristics and in vitro performance. *International journal of pharmaceutics* **2010,** *395* (1-2),243-250. https://doi.org/10.1016/j.ijpharm.2010.05.008

4. Pradhan, S.; Hedberg, J.; Blomberg, E.; Wold, S.; Odnevall Wallinder, I. Effect of sonication on particle dispersion, administered dose and metal release of non-functionalized, non-inert metal nanoparticles. *Journal of nanoparticle research* **2016,** *18* (9),1-14. https://doi.org/10.1007/s11051-016-3597-5
